# Supplementary material for: Genome-Wide Identification and Characterization of Short-Chain Dehydrogenase/Reductase (SDR) Gene Family in Medicago truncatula
Source: Int J Mol Sci. 2021 Aug 31;22(17):9498. doi: 10.3390/ijms22179498 (PMC8430790; doi:10.3390/ijms22179498)
Supplement: Supplementary file 1 [file ijms-22-09498-s001.zip › Table S2.pdf]

>MtSDR1E1

MLNFSRARSQPRAARSMSLGGMDYVDPKKKGNLLGKVFLVAALTALCILVIKRSPSFTTP

SPFSVHEPGVTHVLVTGGAGYIGSHATLRLLKESYRVTIVDNLSRGNLGAVRVLQSLFPQ

PGRLQFIHADLGDAKSVDKIFSENKFDAVMHFAAVAYVGESTLYPLKYYHNITSNTLLVL

ESMAKYNVKTFIYSSTCATYGEPEKMPITEETEQKPINPYGKAKKMAEDIILDFSKNSKM

AVMILRYFNVIGSDPEGRLGEAPRPELREHGRISGACFDAARGIMPGLKVTGTDYNTPDG

TCIRDYIDVTDLVDAHVKALEKAKPGKVGIYNVGTGKGRSVKEFVSACKKATGVDIKVDF

LPRRPGDYAEVYSDPTKIRRELNWTAQYTDLEKSIQVAWKWQKTRPNGYGSS*

>MtSDR1E2

MRPWREGIRNWKEMVSGQYQSSERKTIMVTGGAGFIGSHTVVQLLKQGYTVSIIDNLYNS

VMEAVHRVRHLVGPQLSNNLRFHHVDLRNKQELEMIFSKTKFDDVIHFGGLKAVGESVAE

PLKYFDHNLVGSINLFQVMAKFQCKKMVLSSSAAVYGQPEEIPCVEDFHLQAMNPYGRTK

LFAEEIARDIQKAEPEWRIILLRYFNPVGAHESGEIGEDPRGIPNNLMPYLTQVAVGRLP

ELNVYGHDYPTKDGTAIRDFIHVMDLADGHIAALRKLFATDKIGCGAYNLGTGRGTSVLQ

MVAAFEEASGKKIPIKMCPRRPGDATAIYASTRKAENELGWKAKYGIEEMCRDQWNWASN

NPWGYQLEPGFGNH*

>MtSDR1E3

MNNKTVLVTGGAGYIGSHTVLQLLLGGFKSIVVDNLDNSSEVAIHRVKELAGEFGNNLSF

HKVDLRDRAALEQIFGSTTFDAVIHFAGLKAVGESAQKPLLYYNNNLIGTITLLEVMAAH

GCKKLVFSSSATVYGWPKEVPCTEEFPLSAANPYGRTKLTIEEICRDVHRAEPDWKIILS

RYFNPVGAHPSGYIGEDPRGVPNNLMPFIQQVAVGRRPALTIFGNDYNTVDGTGVRDFIH

VVDLADGHIAALLKLEEADIGCEVYNLGTGKGTSVSEIVRAFEHASGKKIPLVKAGRRPG

DAEIVYASTEKAERELKWKAKYGIDDMCRDQWNWASKNPYGYGPPDSTD*

>MtSDR1E4

MASRVGITNGTSSPLNSSRFLSPSKNSSFQTQNGSNSNSASLMTQNKTVLVTGGAGYIGS

HTVLQLLLGGFKTVIVDNLDNSSEVAVRRVKELAAEFGKNLNFHKVDLRDKAALEQIFSS

TKFDAVIHFAGMKAVGESVQKPLLYYNNNLIGTITLLEVMAAHGCKKLVFSSSATVYGWP

KEVPCTEEFPLSAANPYGRTKLTIEEICRDVNRADPDWKIMLLRYFNPVGAHPSGYIGED

PHGIPNNLMPFIQQVAVGRRPALTVFGNDYKTVDGTGVRDYIHVVDLADGHIAALRKLEE

ADIGCDVYNLGTGKGTSVLEMVRAFEKASGKKIPLVKAGRRPGDAEIVYASTKKAERELK

WKAKYGIDEMCRDQWNWASKNPYGYGSPDSTN*

>MtSDR1E5

MLNLVRSRTQARTTRPTTMGSMEYADPKKKGNFVGKIFLAAALTSLCIIMIKRSPSLKSP

SPFAFHEPGVTHVLVTGGAGYIGSHAAFRLLKDNYRVTIVDNLSRGNLGAVRVLQDLFPE

PGRLQFIYADLGDKKSVNKIFLENKFDAVMHFAAVAYVGESTVDPLKYYHNITSNTLLVL

ESMAKHDVKTLIYSSTCATYGEPEKMPITEVTPQVPINPYGKAKKMAEDIILDFSKNSDM

AVMILRYFNVIGSDPEGRLGEAPRPELREQGRISGACFDAARGIIPGLKVRGTDYKTSDG

TCIRDYIDVTDLVDAHVKALEKAVPAKVGIYNVGTGKGSSVKEFVEACKKATGVNIKVEF

LPRRPGDYAEVYSDPTKINRELKWSAQRTNLEESLRTAWRWQKSHHDGYGIPNVY*

>MtSDR1E6

MVSSSQKILVTGGSGFIGTHTVLQLLQGGFAVSIIDNFDNSVIAAVDRVRELVGPQLSQN

LDFTLGDLRIKDDLEKLFSKTKFDAVIHFAGLKAVGESVANPRRYFDNNLVGTINLYEVM

AKYNCKKMVFSSSATVYGQPDTIPCVEDFKLQAMNPYGRTKLFLEEIARDIQIAEPEWKI

ILLRYFNPVGAHESGKLGEDPRGIPNNLMPYIHQVAVGRLPALNVYGHDYPTRDGSAVRD

YIHVMDLADGHIAAVRKLFATENIGCTAYNLGTGRGTSVLEMVSAFEKASGKKIPLKLCP

RRPGDATEVYASTDKAQKELGWKAKYGVEEMCRDQWNWAKNNPWGYSGKP*

>MtSDR1E7

MSNKTILVTGGAGYIGSHTVLQLLLGGYKVVVVDNLDNSSQKSIDRVKKLAGDFAGNLSF

HKLDLRDRDGLEKIFSSTKFDAVIHFAGLKAVGESVQKPLLYYDNNLIGTIVLFEVMAAH

GCKKLVFSSSATVYGWPKEVPCTEEFPLSAASPYGRTKLYIEEICRDIYRSDPGWKITLL

RYFNPVGAHPSGHIGEDPRGIPNNLMPFVQQVAVGRRSALTVFGTDYSTSDGTGVRDYIH

VVDLADGHIAALCKLDDPKTGCEVYNLGTGKGTSVLEMVAAFEQASGKKIPIVKADRRPG

DAEVVYASTEKAAKELNWKAKYGIDDMCRDQWNWASKNPYGYGESEK*

>MtSDR2E1

MYWVELYVDIHKWVSYISIKAGGLRPWNALSFKAVEHFVVQSECANIIAPYPTGAHPVHW

TPYTCPLTAFKSSAACHKSGYSHTHLYKGDLASTRRNISFHREDSSPPYRSTFCHCCLHR

FLTEQISKPDMASYTPKNILITGAAGFIASHVANRLVRSHPEYKIVVLDKLDYCSNLKNL

LPSKASPNFKFVKGDIGSADLVNYLLITESIDTIMHFAAQTHVDNSFGNSFEFTKNNIYG

THVLLEACKVTGQIRRFIHVSTDEVYGETDEDAVVGNHEASQLLPTNPYSATKAGAEMLV

MAYGRSYGLPVITTRGNNVYGPNQFPEKLIPKFILLAMQGKTLPIHGDGSNVRSYLYCED

VAEAFEVVLHKGEVGHVYNIGTKKERGVIDVAKDICRLFSIDPEANIKFVENRPFNDQRY

FLDDQKLKNLGWAERTTWDEGLKKTIEWYTKNPDWWGDVSGALLPHPRMLMMPGGMERHF

EGSEGENPASVNSSNTRMVVPSTKNTATPQKHPFKFLLFGRTGWIGGLLGKLCEKQGIPY

EYAKGRLEDRASLISDIQNVKPTHVFNAAGVTGRPNVDWCETHKTETIRANVAGTLTLAD

VCREHGILMINYATGCIFEYDAAHPEGSGIGFKEEDKPNFMGSFYSKTKAMVEELLREYD

NVCTLRVRMPISSDLSNPRNFITKISRYNKVVNIPNSMTVLDELLPISIEMAKRNLSGIW

NFTNPGAVSHNEILEMYRDYIDPSFKWQNFTLEEQAKVIVAARSNNEMDGAKLKKEFPEL

LSIKESLIKYVFVPNKKK*

>MtSDR2E2

MYEPKNILITGAAGFIASHVTTRIINKYPSYKIVALDKLDYCSTFKNLQSCTSSPKFKFI

KGDIASVDIVNHVLIEEEIDTIMHFAAQTHVDNSFGNSMEFTYNNIYGTHVLLEACRATN

CVKRFIHVSTDEVYGETDLDANIGNHETSQLLPTNPYSATKAGAEMLVMAYHRSYDLPII

TSRGNNVYGPNQYPEKLVPKFILLAMKGEKLPIHGDGSNVRSYLHSGDVAEAFDVILHKG

VIGQVYNIGTKKERSVLDVAEEICKLFKLDSKKMIEFVHDRPFNDKRYFLDDQKLKKLGW

EERTTWEEGLKMTIDWYRNNPDWWGDVSTALNPHPRFSAINLSDEAQWSFQYGYSRLLRS

FTDVGRREPGLKFLIYGRTGWIGGLLGKICDEERIAWEYGRGRLQDRRSIMEDIKRVMPT

HVLNAAGVTGRPNVDWCESHKAETIKTNVVGTLTLADVCKESDLYVMNFATGCIFEYDKE

HPLGSGKGFKEEDKPNFIGSFYSKTKAMVEELLKEYDNVCTLRVRMPISSDLSNPRNFIT

KISRYNKVVNIPNSMTVLDELLPISIEMAKRNLKGIWNFTNPGVISHNQILELYRDYIDP

SYKWVNFNLEEQAKVIVAPRSNNEMDASKLKNEFPELLSIKDSVIKFVFEPNKKT*

>MtSDR2E3

MIVNFLSRKVSDMATHTPKNILITGAAGFIASHVANRLIRNYPDYKIVVLDKLDYCSNLK

NLIPSRSSPNFKFVKGDIESADLVNYLLITESIDTIMHFAAQTHVDNSFGNSFEFTKNNI

YGTHVLLEACKVTGQIKRFIHVSTDEVYGETDEDAVVGNHEASQLLPTNPYSATKAGAEM

LVMAYGRSYGLPVITTRGNNVYGPNQFPEKLIPKFILLAMQGKVLPIHGDGSNVRSYLYC

EDVAEAFEVILHKGEVGHVYNIGTKKERRVIDVATDVCKLFSIDPETSIKFVENRPFNDQ

RYFLDDQKLKVLGWSERTTWEEGLKKTMDWYINNPNWWGDVSGALLPHPKMLMMPGGMER

HFNGSEEENSAPFVSSTNTRMVVPPTKSIGSSQKPPLKFLIYGRTGWIGGLLGKLCEKQG

IPYEYGKGRLEDRSSLVADIQNVKPTHIFNAAGVTGRPNVDWCESHKTETIRVNVVGTLT

LADVSREHNLLMINYATGCIFEYDEAHPEGSGIGFKEEDTPNFAGSFYSKTKAMVEELLK

DYDNVCTLRVRMPISSDLSNPRNFITKISRYNKVVNIPNSMTVLDELLPISIEMAKRNLR

GIWNFTNPGAVSHNEILEMYRDYIDPNFKWANFTLEEQAKVIVAARSNNEMDASKLKNEF

PELLSIKESLIKYVFEANKKSA*

>MtSDR3E1

MADSSSKTPAPSTTNGDTTPPPRKVALITGITGQDGSYLTEFLLNKGYSVHGLIRRSSNF

NTQRIDHIYVDPHNAHKAHMKLHYADLSDASSLRRWLDTILPDEVYNLAAQSHVAVSFEI

PDYTADVVATGALRLLEAVRSHIDATGRSHIRYYQAGSSEMFGSTPPPQSETTPFHPRSP

YAASKVAAHWYTVNYREAYGIFACNGILFNHESPRRGENFVTRKITRAVGRIKIGLQSKL

FLGNLSASRDWGFAGDYVEAMWLMLQQEKADDYVVATEDSHTVEEFLEVAFGYVGLNWKD

HVVIDKRYFRPTEVDNLKGDASKAKKVLGWKPKVSFEELVRMMVDNDVEMAKKEKVLVDA

GYIDAQQQP*

>MtSDR3E2

MADSSPKTPTPSTTNGDTTPPPRKVALITGITGQDGSYLTEFLLNKGYSVHGLIRRSSNF

NTQRIDHIYVDPHDAHKAHMKLHYADLSDASALRRWLDTILPDEVYNLAAQSHVAVSFEI

PDYTADVVATGALRLLEAVRSHIDATGRSHIRYYQAGSSEMFGSTPPPQSETTPFHPRSP

YAASKVAAHWYTVNYREAYGIFACNGILFNHESPRRGENFVTRKITRVVGRIKIGLQSKL

FLGNLSASRDWGFAGDYVEAMWLMLQQEKADDYVVATEDSHTVEEFLEVAFGYVGLNWKD

HVVIDKRYFRPTEVDNLKGDASKAKKVLGWKPKVSFEELVRMMVDNDVEMAKKEKVLVDA

GYIDAQQQP*

>MtSDR4E1

MGSQNAAAFFDYKSAKVFVSGHRGLVGSAIVRKLTQLGFTNLILRTHTELDLTRQSDVEA

FFASTKPEFVIVAAAKVGGIHANNTYPADFIAINLQIQTNVIDSAYRNGAKKLLFLGSSC

IYPKFAPQPIPEDALLTGPLEPTNEWYAIAKIAGIKMCQAYRIQHKWDAISGMPTNLYGP

NDNFHPENSHVLPALMRRFHEAKVNGAKEVVVWGTGSPLREFLHVDDLADAVVFMMEKYS

GVEHLNVGSGKEVTIKELAESMKEVVGFEGDLVWDSTKPDGTPRKLMDSSKLAALGWTPK

VSLKDGLVDTYKWYLENVKQ*

>MtSDR6E1

MKQLHKQQSLNHRREEEMGSETPPYSPKSMKHTRTLPRSINYLLREQRLLFILVGILIGS

TFFIIQPTLSRISPPEAGLFLPRSGLVRFNTGNEGASLRVGRIPAGIGGRRLRVVVTGGA

GFVGSHLVDKLIGRGNDVIVIDNFFTGRKENLVHLFGNPRFELIRHDVVDPILLEVDQIY

HLACPASPVHYNVGVRTFVFVVVQWLFGISNFVIGFLDPSLYYVIGDLQDKCTNVMGTLN

MLGLAKRIGARFLLTSTSEVYGDPLEHPQKETYWGNVNPIGERSCYDEGKRTAETLAMDY

HRGAGVEVRIARIFNTYGPRMCLDDGRVVSNFVAQAIRKQPLTVYGDGKQTRSFQYVSDL

VNGLAALMDGEHVGPFNLGNPGEFTMLELAQVVKETIDSSATIEYKQNTADDPHMRKPDI

SKAKELLNWEPKVPLREGLPLMVSDFRNRILNEDEGKGMR*

>MtSDR6E2

MSSELTFRGHETQQVNDEYSPKPNKPWLSVIRPIRYMLREQRLVFVLIGIVIASVFFTII

PSSSTSSSSSFSTRPYESDSISYFDRESKTTPAVYKQRAASVVHSSGKIPLGIKRKGLRI

VVTGGAGFVGSHLVDRLMARGDSVIVVDNFFTGRKENVMHHFGNPRFELIRHDVVEPLLL

EVDQIYHLACPASPVHYKFNPTNVVGTLNMLGLAKRVGARFLLTSTSEVYGDPLEHPQKE

TYWGNVNPIGVRSCYDEGKRTAETLTMDYHRGAGVEVRIARIFNTYGPRMCLDDGRVVSN

FVAQALRKEPLTVYGDGKQTRSFQYVSDLVEGLMRLMEGEHVGPFNLGNPGEFTMLELAK

VVQETIDPDAKIVYRDNTEDDPHKRKPDISNAKEHLGWEPKVDLRKGLPLMVSDFRQRIF

GDHKEGGSDA*

>MtSDR6E3

MAANSSNGDQHNGDQQTTTKQPPLPSPLRFSKFFQSNMRILVTGGAGFIGSHLVDRLMEN

EKNEVIVADNYFTGCKDNLKKWIGHPRFELIRHDVTETLLVEVDRIYHLACPASPIFYKY

NPVKTIKTNVIGTLNMLGLAKRVGARILLTSTSEVYGDPLIHPQPETYWGNVNPIGVRSC

YDEGKRVAETLMFDYHRQHGLEIRIARIFNTYGPRMNIDDGRVVSNFIAQAIRGEPLTVQ

LPGTQTRSFCYVSDMVDGLIRLMEGENTGPINIGNPGEFTMTELAENVKELINPAVEIKM

VENTPDDPRQRKPDITKATELLGWEPKVKLRDGLPLMEEDFRLRLGVPRKN*

>MtSDR6E4

MAGNSSNGDQSTAKQPPLPSPLRFSKFFQSNMRILVTGGAGFIGSHLVDRLMENEKNEVI

VADNFFTGSKDNLKKWIGHPRFELIRHDVTETLMIEVDQIYHLACPASPIFYKYNPVKTI

KTNVIGTLNMLGLAKRVGARILLTSTSEVYGDPLEHPQPESYWGNVNPIGVRSCYDEGKR

VAETLMFDYHRQHGIEIRVARIFNTYGPRMNIDDGRVVSNFIAQALRGESLTVQAPGTQT

RSFCYVSDLVDGLIRLMGGSDTGPINLGNPGEFTMTELAETVKELINPNVEIKIVENTPD

DPRQRKPDITKAKELLGWEPKVKLSEGLPLMEGDFRLRLGVDKKE*

>MtSDR6E5

MSSELIHRNQTRDQTTDSSSHRDNPLPRTRTNIINMLLNRVPFLLIGIAISTFFFHYLPS

RSTLPQHHDSSSFVGTELTLPTRRVLLEEHGRDERKRRVPLAVGLKSKRQKRVLVTGGAG

FVGSHLVDRLIERGDNVIVIDNYFTGRKENVVHHIGNPNFELIRHDVVEPILLEVDQIYH

LACPASPVHYKFNPTNVVGTLNMLGLAKRVGARFLLTSTSEVYGDPLQHPQAETYWGNVN

PIGVRSCYDEGKRVAETLAMDYHRGAGIEVRIARIFNTYGPRMCIDDGRVVSNFVAQALR

KDPLTVYGDGKQTRSFQFVSDLVEGLMRLMEGEHVGPFNLGNPGEFTMLELAQVVQETID

PNAKIEFRANTEDDPHKRKPDISKAKELLGWQPSVSLREGLPLMVADFKQRLFGDGDKGA

AAA*

>MtSDR7C1

MVGIFSLVTGMAGPSGFGSSTTAEQVTQGIDASNLTAIITGGASGIGLETARVLALRKVH

VIIAARNMESAKEAKQIILQDNESARVDIMKLDLCSVKSVRSFVENFLALDLPLNILINN

AGVMFCPFQLTQDGIEMQFATNHLGHFLLTNLLLEKMKQTAKATGIEGRIINLSSIAHTY

TYEEGIRLDNINDQIGYSDKKAYGQSKLANILHANELSRRLKEEGVNITANSVHPGVIMT

PLMRHSSLLMNFLKMFTFYIWKNVPQGAATTCYVALHPSLKGVTGKYFLDCNEFQPSAFA

SNGLLGRKLWDFSNKLINSISKS*

>MtSDR7C2

MWPFSKKGVSGFSWNSTAEQVTHGIDATGLTAIVTGASSGIGAETARVLALRGVHVIMGV

RNLVAAKDVKDTILKDIPSAKLDAMELDLSSLDSVKKFASEYNSSGRPLNILINNAGVMA

CPFKLSKDNIELQFATNHIGHFLLTNLLLDTMKKTTRKSKKEGRIVNVASEAHRFAYSEG

IRFDKINDESSYSRWGAYGQSKLANILHANQLTKHLKEDGVDITSNSLHPGTIVTNLFRH

NSAVNGLINVVGRLVMKNVQQGAATTCYVALHPQVKGVSGEYFSDSNVYKTTPHGRDADL

AKKLWDFSINLVKQK*

>MtSDR7C3

MWPFSRKGVSGFSWKSTAEEVTHGIDATGLTAIVTGASSGIGTETTRVLALHGVHVIMAV

RNKVNANNTREAILKEIPSAKIDVMELDLSSLESVKKFASEFNSSGLPLNILINNAGVMA

CPFMLSNDNIELQFATNHLGHFLLTNLLLDTMKKTASESKTEGRIVNVSSEAHKFAYSEG

IRFDKINEQSSYSKWGAYGQSKLANILHANQLTKHFKDDGVNIIANSLHPGGIATNLYRH

NSAINGIVNVVGKLVMKNVPQGAATTCYVALHPQVKGVSGEYFSDNNVAKSSSHGKDADL

AKKLWDFSMNLIKDK*

>MtSDR7C4

MWPFSKKGISGFSGSSTAEQVTHGIDAIGLTAIVTGASSGIGVETMRVLALRGVHVIVAV

RNKVAANDIKEAILKEIPSAKIDVMELDLSSLESVKKFASEFNSSGLPLNILINNAGIML

CPFMLSKDNIELHFATNHLGHFLLTNLLLDTMKKTAHQSKKEGRIVNVSAKAYIFAYPEG

IRFDKINDQSSYSKWGAYGQSKLANILHANELARRLKDDGVDIIVNSLHPGIILTNLSRH

MSVIDGIIKVIGKLVMKNVQQGAATTCYVALHPQVKGVSGEYFSESNVAKLRSQGRDADL

ANNLWDFSMNLIKDK*

>MtSDR7C5

MWPFSKKGVSGFSGNSTAEKVTHGIDASGLTAIVTGATSGIGVETTRVLALRGVHVIMAV

RNKVAANDINEAILKEIPSAKVDVMELDLSSLESVKKFASEFNSSGLPLNILINNAGIMS

CPFMLSKDNIELQFATNHLGHFLLTNLLLDTMKKTAHQSKKEGRIVNVSGEAYTFAYSEG

IRFDKINDQSSYNKQSAYGQSKLANILHANELARRLKDDGVDIIVNSLHPGIILTNLSRH

MGVIHGIIQVIGKLVMKNVQQGAATTCYVALHPQVKGVSGEYFSDNNVAKRESQGRDADL

AKKLWDFSMNLITKE*

>MtSDR7C6

MAGFCSLPNSCHNKITVGMINLIKDLLRSIYFLCSIQFLRMALLWTFSVAFSHYQLFKDS

LFSHKIVSYPRSSPSTFPNKPVCVITGATSGLGLSTACKLSKEGYVVVIVGRSEQLLSEA

ITKIKGWNEDAHLKAFQADLSSVESIIKFSTSLRQWLLDSDLHCSVQILINNAGILATSP

RVTTEGYDKMIATNYIGPFVMTKLLLPLLESSPVSSKIVNVTSFTHRAVTNMQVDEGTVS

GKRFLKSKQYPYAQIYEYSKLCLLLFSYELHRQLCQMGKSHQIFVNVADPGVVQTNIMRE

VPASLSWVAFFVLKRLRLLESFESGNDSIIDAALTPPGTSGVYFFGGKGRTINSSALSQD

TKLAHELWETTSDLLSVTPFGNKRNNF*

>MtSDR7C7

MTITKIKGWNEDVHLKAFQADLSSVESIIKFSTSVRQWLLNSDLHCLVQILINNAGILAT

SPRITTEGYDYKNYIGPFVMTKLLLPLLERSHVSSKIVNVTSFTHRVDEGIIYGKRFLKS

KQYPYAQIYEYSKLCLLLFSYELHRQLFQIGKSHQIFVKLRSVMRPSFVKDTMKLLSEYL

SPNKEHTLPLKLQLPN*

>MtSDR7C8

MKATLRYLAGIAGPSGFGSNSTAEQVTQNCSSFLPSGLTALITGGTSGIGAETARVLAKR

GVRIVIGARDLKKAMKVRDNIQKESPNAEVILLEIDLSSFCSVQRFCSDFLALDLPLNIL

INNAGVFSQDLEFSAEKIEMTFATNYLGHFLLTEMLLDKMIETSKKTDIQGRIINVSSVI

HSWVKRHGFCFKDILNGKNYNGTRAYAQSKLANILHAKEIARQLKARKARVTMNAVHPGI

VKTGIIKSHKGLITDSLFFIASKLLKSTSQGAATTCYVALSPKTEGVSGKYFTDCNESKC

SRLANEESEAQKLWNNTHALIHKRLHQATNNM*

>MtSDR7C9

MTLLWTFSVAFSNYQLFKDSLFSHKIVSYPRSSPSTYPNKPVCVITGATSGLGLSTACKL

SKEGYVVVIVGRSEQLLSEADLSSVESIIKFSTSLRQWLLDSDLHCSVQILINNAGILAT

SLRVTAEGYDQMIGTNYIGPFVMTKLLLPLLESSHVSSKIVNVTSFTHRAVTNMQVDEGT

VYGKKFLKSKQYPYAQIYEYSKLCLLLFSYELHRQLCQMGKSHQIFVNVANPRVVQTNIM

REVPASLSWVAFFVLKRLRLLESSECGNDSIIDAALVPPGTSGAYFFWGKGRTINSSALS

QDAKLAHELWETTSNLLSVTPFGN*

>MtSDR7C10

MFGIKSKRESSMKETIRYLAGIAGPSGFGSNSTAEQVTQHCSLFIPSNLTALITGATSGI

GAETARVLAKRGVRVVVGARDMKKAMKVKEKIQEESPYAEVILLEIDLSSLASVQRFCSE

FLALELPLNILINNAGVYSHNLEFSEEKIELTFATNYLGHFLLTKMLLEKMIDTANKIGI

QGRIINISSVIHSWVKRSCFCFKDMLTGKNYNGTRAYAQSKLAMILHVKEMARQLKARNA

RVTINAVHPGIVKTGIIRAHKGLITDSLFFIASKLLKTTSQGASTTCYVALSQKTEGVSG

KFFTDCNESSCSRLANDESEAKKLWNNTNNLLHKRLRQAAIGSSSSLYS*

>MtSDR7C11

MSPSEFTLSSSAEEVIPSEFTLSSTAEEVTHGIDGSGLAAIVTGTTHGIGIETARVLALR

GVHVIMGVRNINAGKIVREEILKEIPKAKVDVMELDLSSMASVRKFASEFNSSSLPLNIL

INNAGICAPPFTLSKDNIELQFATNHLGHFLLTNLLLDTMKKTANESKKEGRIINVSSDG

HGYTYDEGILFDNINDESSYQRWRAYGQSKLANILHANELARNFKEDGVGITANSLHPGC

IGTNIVSREVGQTMPLDLRKSLGKIVVKTIQQGAATTCYVALNPKVKGISGKFFCDSNVA

EPSSLGKDTDLAKKLWDFSMNLIK*

>MtSDR7C12

MTSSGFSLSSTAEEVTHGIHGSGLTAIVTGTTHGIGIETARVLALRGVHVIMGVRNINAG

KVVREEILKEIPKAKVSVMELDLSSMASVRKFASEFNSSSLPLNILINNAGICAAPFTLS

KDNIELQFATNYLGHFLLTNLLLDTMKKTANESKKEGRIIIVSSDGHNYTYPEGILFEKI

NDESSFDRWKAYGQSKLANILHANELARYLKEDGVNITANSLHPGAIITNIVSPEVGQTI

PKGLLNVLGEFVIKSVQQNRKVGCYYESRRNFRNMMEVAGDEAGSIPTELCGRPA*

>MtSDR7C13

MALLWTFSVAFSHYQLFKDSLFSHKIVSYPRSSPSTFPNKPICVITGATSGLGLSTACKL

SKEGYVVVIVGRSEQLLSETIKKIKGWNEDAHLKAFQADISSAESIIKFGTSLRQWLLDS

DLHCSVQILINNAGILATSPRVTTEGYDKMIATNYIGPFVMTKLLLPLLESSPVSSKIVN

VTSFTHRAVTNMQVDEGTVSGKRFLKSKQYPYAQIYEYSKLCLLLFSYELHRQLCQMGKS

HQIFVNVADPGVVQTNIMREVPACLSWVAFFVLKRLRLLESFESGNDSIIDAALTPPGTS

GVYFFGGKGRTINSSALSQDTKLAHELWETTSDLLSVTTFGNKRNNF*

>MtSDR7C14

MVGVISLITGMAGPSGFGSASTAEQVTQGIDASNLTAIITGGASGIGLETTRVLALRKVH

VIIAARNIESAEEAKQQITQENKSARVDIMKLDLCSTKSVRSFVDNFIALDLPLNILINN

AGIMFCPFKISEEGIEMQFATNHLGHFLLTNLLLDKMKQTAKTTGIEGRIINLSSIAHRY

TYFRKGIKFEKINDKKGYSSKKAYGQSKLANILHANELSRRLQEEGVNITVNSVHPGVIM

TPLMRYSSYTMHLLKIFSFYIWKNVPQGAATTCYVALHPSVKGVTGKYFVDCNEFKPSAY

AKNKLLAKKLWDFSNKLINSISKA*

>MtSDR7C15

MWFIGWKGPSGFSASSTAQQVTHGIDGTSLTAIITGASSGLGLETTRILAFRGVHVVMAV

RNVKNCIDIKESILKEIPAAKIDVFELDLSSLASVRKFAADFNSSGLPLNILINNAGLMA

TPFMLSQDNIELQFATNHLGHFLLTNLLLETMKKTVRECNQEGRIVIVSSEAHRFAYSEG

IRFDKINNESEYSSYFAYGQSKLANILHANELSRRLKEEGVQITVNSLHPGTIVTNILRH

HGYFNAVANMVGKYFLKNVQQGAATQCYLALHPQVKGISGEYFTDSNKASPTSLAKDTKL

AQKLWELSVSLSNTK*

>MtSDR7C16

MLETIKYLIGSAGPSGFGSKSTAEQVTETCGDLRSITAIITGGTSGIGAETARVLAKRGA

RVILPARSMKNAEETRGRIVTECPEAEIIVMALDLSSVNSVTNFVTRFHSIGFPLNLLIN

NAGKFAEEHAISEDGVEMTFATNYLGHFVLTKMLMKKMVETAKETGIEGRIVNVSSAIHG

WFTGDVISYLAHICRNKSEYDATRAYALSKLANVFHTNELARRLKEMDANVTVNCVHPGV

VRTRLTREREGFLTDLVFFLASKLLKTIPQAAATTCYVATHPRLVNVSGKYFADCNETST

SKLGSNITEASRLWAVSELMISKGPKAAFDLINNLEF*

>MtSDR12C1

MDLPHNFFLLATALLGFISVCKFFFHFFNWIWIMFLRPHKKLIDYGSWAIITGSTDGIGK

AMAFEFALKGLNILLVGRSPLKLEATSKEIIDKTFGNVEVKSVVVDLQNNSGEEIMNKVE

EAIDGLDVGILVNGAGVAYPYARFFHEVDLDLMDTIIKVNVEGTTWITKAVLPSMIKNKK

GAIINIGSGSTVVIPSYPLVTLYAASKAYLAMFSACTNLEYKQLGIDIQCQVPLFVSTKM

TRMRASLFVPTPDKYSKACTKWIGYEKLVVPYLFHNLQSFLIRKIPDVFLDSYMLRNLLY

MRKRGLIKDSQIKGSKASSNSKTN*

>MtSDR12C2

MDCCIISKLKTQPFWFLILFSLGLFTILRFTLLLLNWFYVNFLRQPKNLKNYGSWALVTG

PTDGIGKSFAFELARKGLNLILVGRNPEKLKDVSDSIKAKFGKTEVKTVVVDFTGDLDDG

VKRIVETIDGLDVGVLINNVGISYPYARFFHEVDQELLKNLIKVNVVGTTKVTQAVLPGM

LKRKKGAIVNIGSGAAIVIPSDPLYAVYAATKAYIDQFSRCLYVEYKKSGIDVQCQVPLY

VATKMASIRRSSFFVPSTDGYAKAGVKWIGYEPRCTPYWPHTLLWAVARSLPESIVDTWR

LGFCMGIRKRGQLKDSKKQE*

>MtSDR12C3

MHQLEVLIIFLPFLLGLTLTFNCFVTLITWIFNSCLRSDTHLIKTYGSWALITGATDGIG

KALAYQLAQKSLNLILVSRNSKKLETVKNEIKTKYPRIDIKTITIDFSEDFTESLWEIEV

LASDLNLGILINNVGITYPKAMFFHEVKEEMWMKIVSVNIESTTRITKAVLGGMMERKKG

AIVNIGSGAAVVVPSHPLFTIYAATKAYVDQFSRSLYMEYKQYGIHVQCQVPLYVATNMV

SRVASIERDSLFIPTPEGYARASIRKIGYETRCTPYWAHSIQWAFARLIPDPLLDYWRMS

IGLRRRSSHKNKD*

>MtSDR17C1

MVASTESPFKPDILKGKVALITGGASGIGFEISTQLGKHGASVAVMGRRKQVLQSAVSVL

QSLSIPAVGFEGDVRKQEDAARVVDLTFKHFGRIDILVNAAAGNFLAAAEDLSPNGFRTV

LDIDSVGTFTMCHEALKYLKKGAPGRNSSSGGLIINISATLHYGASWYQIHVSAAKAAVD

STTRNLALEWGTDYDIRVNGIAPGPIGETPGMSKLAPEEIGSRGRDEMPLYKLGEKWDIA

MAALYLASDAGKYINGDTMVVDGGLWLSRPRYLPKEAVRQVSREVEKRSRNEPIGVPKSK

L*

>MtSDR22E1

MQAITRRLGHESLKSSTLLKSSYPISDHYYGVNHERYVSTIATKGVGHLVRKGTGGRSSV

SGIVATVFGATGFLGRYVVQQLAKMGSQVLVPFRGSEDSPRHLKLMGDLGQIVPMKFNPR

DESSVKAVMARANVVINLIGRDYETRNFSYEEVHYHMAEKLAKISKEHGGIMRFIQVSCL

GASPSSSSRMLRCKAAAEEAVLRELPEATIFKPAVMIGTEDRILNRWAHFAKKYGFIPLM

GNGNTKIQPVYVVDVAAALTTALKDDGTSMGKIYELGGPEIFTVHQLAEIMYDVIREWPR

YVNVPLPIAKALATPRELFLNKLPFPLPKPEMFNLDQIHAYAADTVVSENALTFNDLGIV

PHKLKGYPIEFLIQYRKGGPQFGSTISEKVSPDAWP*

>MtSDR25C1

MEIPKRFKGKVAIVTASTQGIGFTIAERLGLEGASVVISSRRQKNVDVAAEKLRAKGIDV

FAVVCHVSNALQRKDLIDKTVQKYGKIDVVVSNAAANPSVDSILQTQDSVLDKLWEINVK

ATILLLKDAAPYLPKGSSVVIISSIAGYHPPASMAMYGVTKTALLGLTKALAGEMAPKTR

VNCVAPGFVPTNFASFITSNSAMREELEAKTLLGRLGTTEDMGAATAFLASDDASYITGE

TIVVSGGMPSRL*

>MtSDR31E1

MAAAEEGWCVVTGGRGFAARHLVEMLIQLNTYCVRIADLGSTIELEPSEQLGLLGQALHS

GRAQYVSVDLRNKPQLLKAFDGVEVVFHMAAPNSSINNYQLHHSVNVEGTKNVIDACVEL

KVKRLIYTSSPSVVFDGIHGIHNGSESLPYPPSHNDHYSATKAEGEGLVIKANGTSGLLT

CCIRPSSIFGPGDKLLVPSLVDAAKAGKSKFIVGDGNNVYDFTYVENVAHAHICADRALA

SEGTVSEKAAGEAYFITNMEPIKFWEFMSLILEGLGYQRPSIKIPVFVIMPIAHLVEWIY

RLLGPYGMKVPQLTPSRIRLTSCTRSFDCSKAKDRLDYAPIIPLQEGIRRTIESYPHLRA

ENQLKSKREGPSKASVYLGSGRVADTLLWKDKKQTFTTLLVFIAIYVNFIASENTFITAL

TKLLLYSSIGLFIHGILPAKILGYTVEKMPTSWFHLSEDISNQIAFSVASSWNFAVNALK

SLAEGNSWVMFFKVVFTLLVLSFLGAFSLKNLYTIGLTLGFTAFYVYEKKEEDIDGIFLK

SHTFGCKLISDLTKKFVTSKID*

>MtSDR31E2

MAVNADRFQDSNPKTCVVLGGRGFIGKSLVLQLLKLGNWIVRIADSTHSLNLHHSESLLA

EALSSSRASYFHLDLTDKHRIAKVLEGSSVVFYFDVDSSNNGDHFCSLYKLIVQGAKNVI

IACRESKVKRLIYNSSADVVFDRDKPLAYPWKVDNMLIDLKAQAEALILNANDIDGVLTC

SLRSSNVFGPGDSELVPFFLKLARYGFTKFIIGTGDNLTDFTFSENVAHAHICAEEALNF

QTVSVAGKAFFITNLEPMKFWKFLSLLLEGLGYRRPFIKLPANLVQYVLSVLKWLYEKSG

PGYFNYPLLVHFIQLALHTRTFNCSAAQKYIAYAPIVSLEEGVTLTIESFSHLAKDSSFS

RCCDRSKADKLLGSGKVADILLWRNEKASFTCFLGLVFLFYWFFLSGSTFISSAARLLLF

ATLLLCGHGFLPSKLFGFSIQRVPGSNFKISDTAVKDSVTITLHLWNKGFQNIKGLAQGD

DWSIFFKVAGFLYLLKLFLSKLLTTLIGVGLVFAFMVFFVYEQYESEIDGLVDILITISK

EFMVYLMRNLPVSVSRLLHYGDNFQHYQGPECGKDLR*

>MtSDR34C1

MLVLFLFIFLLLLFLFKFATAYGDFTLMSKKKPKHELIEDKVFWITGASRGIGEILAQQL

ASLGAKLILSARDEADLNRVKSQLKGKHADEAKILPLDLTSGEDSLRKVVDEAESLFPDS

GVDYMIHNAAYERPKSSVLDVTEESLKATFDVNVFGTITLTRLLTPFMLRRGKGHFVVMS

SAAGKTPAPGQAVYSASKYALNGYFHSLRSELCQKGIQVTVVCPGPIETANNSGSQVPSE

KRVSAEKCVELTIIAATHGLKEAWISYQPVLAVMYLVQYMPTIGYWLMDKVGKNRVEAAK

EKGNAYSLSLLFGKKKAV*

>MtSDR35C1

MADAFFSFFILLPLLLLPLLYFLVRPRPIKIPITNRHVFITGGSSGIGLALAHRAAADGA

RVSIMARSLQKLEEAKNSVKNSTGVEVAIFAADVRDYDAVKKAVDEAGPIDVLLLNHGVF

YALELEKMELSDVKFTIDVNLMGCLNMIKAALPQMKNRKDTLPASIAFVSSQAGQVGIYG

YVAYSASKFGLRGLAEALQQEVIGDNIHVSLIFPPDTDTPGLVEENKRKPELTKIIAASS

GFMKADEVAQKAFDGIRRGSFIISCNLEGIALSLATSGLSPQRSFLMAFVEVIAAGIMRI

AALCFQWNWYGSIEKWHKQRKLFLAKLKAFSNYKDPHGNCRTKVANFKLGYRINFEHDYL

SVHLKALDAL*

>MtSDR35C2

MVFGSIQIIYADSTSFGQLNLKKKRIAYSSVSHMGFIILGIGSISDIGLNEAVLQIISHG

LIGATLFFLGMAIPMPKIFTIFTILSMTSLALSGMSGFVAELIVFFGIIASQKYLFMMKI

LSFINGGRILHLLPLLLLPLLYFLVRPRPIKIPITNRNVFITGGSSGIGLALAHRAAADG

ARVSIMARSLTKLEEAKNSVKHATGVEVRIFDAVKKVADEAGPIDVLLLNHGVFYALELS

DVKFTIDVNLMGCLNMIKAALPHMKKNRKDTLPASIALFHHKLVGIYGYVAYSASKFGLR

GLAEALQQEVIGDNIHVSLIFPPDTDTPGLVEENKRKPELTKIIAASSGFMKADEVAQKA

FDGIRSGSFIISCNLEGIALSLATSGLSPQRSFLMAFVEVIAAGIMRIAALCFQWNWYGS

IEKWHKQRKYTSKHLMHSELYTHSVKCKTNVVFIHSALR*

>MtSDR40C1

MKKSGTLQLQLKFHFSFNIVSLTMFLVKTWRQTAFGVYGYMNFTKPAFLEHSKNFKPEET

EVQIPGKNCIVTGANSGIGYAAAEGLAQRGATVYLVCRNKERGEAALSQIQTKTGNQNVY

LEICDLSSVTDIKSLASRFSEKNVPVHVLVNNAGLLEQNRVTTSEGFELNFAVNVLGTYA

MTELMVPLLEKASPNARVITVSSGGMYSTPLTNDLQYSESSYNGTLQYARNKRVQVALTE

NWGETYKNKGIGFYSMHPGWADTPGVAKSLPGFSKSLAGKLRTSEEGADTVIWLALQPKE

KLVSGAFYFDRAEAPKHLSYNATSGSHTLINSVIDSLRSLASLSA*

>MtSDR42E1

MHLSENEGIEGKSFVVTGGLGFVGSALCLELIRRGAQQVRAFDLRQSSPWSHLLKLKGVN

CIQGDVTRKEDVERVLRGADCVFHLAAFGMSGKEMLQFGRVDEVNINGTCHILDACIDLG

IKRLVYCSTYNVVFGGQKILNGNEALPYFPIDRHVDPYSRSKSIAEQLVLKNNARTLKND

TRNHLYTCAVRPAAIYGPGEDRHLPRIITMARLGLLLFRIGDKTVKSDWVFVDNLVLALI

MASMGLLDDNNDKGKRPIAAGQAYFICDGSPVNSFEFLQPLLRSLDYDLPKRSLALEHAL

VLAKICQGVYTILYPLLNRWWLPQPFILLPSEALKVGVTHYFSYLKAKEELGYVPMVTSR

EGMDSTISYWKQRKRQILDGPTIYTWLFCVVGMTSLFCAGFLPDMGIMFLLRAICLFVFR

SMWMTRLVFIIATAVHFIEAIYAWYLAKRVDPVNARGWFWQTFALGFFSLCFLLKRARE*

>MtSDR50E1

MDIPSSPGKFKMDKRTRWHKSLPKLAFWSILFFGVIYFLFFRTPSSFSSDTSRRSLRTYN

YGGAAWEKKVRSSAKVTSKNGVSVLVTGAAGFVGSHVSIALKRRGDGVVGIDNFNDYYDP

MLKLGRQALLERTGVFIVEGDINDPTLLKKLFEVVPFTHVMHLAAQAGVRYAMENPGSYV

HSNIAGFVNLLEVCKSAEPQPAIVWASSSSVYGLNSKVPFSEKDRTDQPASLYAATKKAG

EEIAHTYNHIYGLSLTGLRFFTVYGPWGRPDMAYFFFTKDILRGKSITIFEGPNHGTVAR

DFTYIDDIVRGCLGALDTAEKSTGSGGKKRGTAQLRVFNLGNTSPVPVTDLVSILERLLK

VKAKRNVMKMPRNGDVQFTHANISYAKKEFGYKPTTDLQTGLKKFVRWYVNYYSAGKKAD

E*

>MtSDR50E2

MASPPDTSKTIKLERYNSYIRKVNSTKLLNASSKLLFRATLLIALVLVFFFTFNYPPLSD

STNHHFHTHSHFLTSAFGGGGAWERHVRHSAIPRRPNGFTVLVTGAAGFVGSHCSLALKK

RGDGVIGLDNFNSYYDPSLKRARQALLTQHQIFIVEGDLNDAPLLTKLFDVVPITHILHL

AAQAGVRYAMQNPQSYIKSNIAGFVNLLEVSKAANPQPAIVWASSSSVYGLNTENPFSEL

HRTDQPASLYAATKKAGEEIAHTYNHIYGLSLTGLRFFTVYGPWGRPDMAYFFFTKDILH

GKTIDVYQTQEGKEVARDFTYIDDIVKGCVGALDTAEKSTGSGGKKKGPAQLRIYNLGNT

SPVPVGKLVSILENLLSTKAKKHIIKMPRNGDVPYTHANVTMAYKDFAYKPTTDLATGLR

KFVKWYVRYYGIQSRLKKENELPEDSA*

>MtSDR50E3

MSQLKQMSHADNSAPSTPGKFKMEKASYFNRVRWHASPAKLCLWSFVFSAAILIFFFRSP

ASSPLPADPSRRSLRSPSNWGGPVWEKRVRSSARVRSRNGFSVLVTGAAGFVGTHVSAAL

KRRGDGVLGIDNFNDYYDPSLKRARQALLERTGVFIVEGDINDAALLRKLFEVVPFTHVM

HLAAQAGVRYAMENPGSYVHSNIAGFVNLLEVCKSVNPQPSIVWASSSSVYGLNTKVPFS

ERDRTDQPASLYAATKKAGEEIAHTYNHIYGLSLTGLRFFTVYGPWGRPDMAYFFFTRDI

LKGKTIPIFEAANHGTVARDFTYIDDIVRGCLGALDTAEKSTGSGGKKRGPAQLRVFNLG

NTSPVPVSDLVGILERLLKTKAKRNIMKLPRNGDVQFTHANISYAQRELGYKPVTDLQAG

LKKFVRWYLNYYSSGKKAVE*

>MtSDR50E4

MAQQVNNETTPSTPGKLKPDKPHHRFRIHPPHSRYTFICILLSAFLVFLLFSTFNPPPPS

TTAPRRVLGDSWGGSHWERLVSKSTRRNSASNKIVLVTGSAGFVGSHVALALKRRGDGVL

GIDNFNRYYDVNLKHARQKLLERAGIFVVEGDINDGTLLKKLFDVVPFTHVMHLAAQAGV

RYAMQNPNSYVHSNLAGFTVLLEACKSANPQPAIVWASSSSVYGLNSKVPFSEKDRTDQP

ASLYAATKKAGEGIAHTYNHIYGLSITALRFFTVYGPWGRPDMAYFFFTKDILKGKQITV

FESPDGGSVARDFTYIDDVVKGCLGALDTAKKSTGSGGKKKGSAQFRVFNLGNTSPVPVS

KLVAILEKLLKVNAKKRVLPMPRNGDVKFTHANISLAQREFGYKPTIDLETGLRKFVKWY

LEFYSSGSIKKGSSSW*

>MtSDR50E5

MPSSSSLEDQLYPSTPGKFKIERNHGLMNRQVHRCFGSSSTMFLWALLLIASTVSYVTFQ

GVVDSGSRYLSASWGGIQWEKQVRASAQIHRQGGMSVLVTGAAGFIGSHVSLALKRRGDG

VVGLDNFNDYYDPSLKKARKALLQSRGVFIVHGDLNDAKLLAKLFDVVAFTHVMHLAAQA

GVRYAMENPMSYVNSNIAGLVTLLEACKTANPQPSIVWASSSSVYGLNEKVPFSEMtSDRTD

QPASLYAATKKAGEEITHTYNHIYGLSITGLRFFTVYGPWGRPDMAIFTFTRNMLQGKPI

TVYRGKNRVDLSRDFTYIDDIVKGCVGSLDTSGKSTGSGGKKRGAAPYRIFNLGNTSPVT

VPTLVSILERLLKVKAKKNIVDMPGNGDVPFTHANITSARREFGYKPTTDIQTGLKKFVK

WYLSYYGYGKTTLN*

>MtSDR50E6

MGGIGILPSKPEKYHYQPYNNINNNNILRIRSSKFTLCSSIFVFLAFIIFFFILSPSTSS

LPTKNSWGGPEWEKRVTKSTRHNSPSGSPLTVLVTGASGFVGMHVSLALKRRGDGVLGID

NFNRYYDINLKRTRAKVLSRAGVFVVEGDINDVHLLRKLFDVVAFTHVMHLAAQAGVRYA

MRNPNSYVHSNIAGFVNLLEVSKSANPQPAIVYASSSSVYGLNSKTPFSEKDRTDQPASL

YAATKKAGEEFAHTYNHIYGLSVTGLRFFTVYGPWGRPDMAVYLFTKDILKGKQITVFES

PDGGSVTRDFTYIGDIVKGCLGALDTAKKSTGSGGKKKGNAQYRIFNLGNTSPVPVSELV

NILEKLLKVNVKRKVVPMPINGDVRFTHANISRAQRELGYMPTTDLEAGLKKFVRWYLDF

HSPLKNKNVW*

>MtSDR52E1

MAQLLSSSCSLTFSASNKPCLKPFHQCSTSFSNTVVCDNSKTPFRQLFLREQKPRKSLAV

VNASTISTGQEAPVQTSSGDPFKPKRVMVIGGDGYCGWATALHLSNKGYEVAIVDNLVRR

LFDHQLGLDSLTPISSIQDRIQCWKSLTGKSIELYIGDICEFEFLSETFKSYEPDAVVHF

GEQRSAPYSMIDRSRAVYTQQNNVVGTLNVLFAIKEYREDCHLVKLGTMGEYGTPNIDIE

EGYITITHNGRTDTLPYPKQASSFYHLSKVHDSHNIAFTCKAWGIRATDLNQGVVYGVRT

DETAMHEELCNRFDYDAIFGTALNRFCVQAAVGHPLTVYGKGGQTRAFLDIRDTVQCVEL

AIANPANPGEFRVFNQFTEQFKVTELAELVTKAGEKLGLDVKTISVPNPRVELEEHYYNC

KNTKLVDLGLKPHFLSDSLIDSLLNFAVQYKDRVDTKQIMPGVSWRKVGVKTKTLTS*

>MtSDR57C1

MASGEQKFPPQKQDTQPGKEHVMDPLPQFTCPDYKPSNKLQGKVAVITGGDSGIGRAVCN

LFSLEGATVAFTYVKGDEDKDAKDTLEMLRNAKSADAKDPMAVAADLGFDENCKKVVDEI

VNAYGHIDILVNNAAEQYECSSVEEIDESRLERVFRTNIFSYFFMTRHALKHMKEGSSII

NTTSVNAYKGNAKLLDYTSTKGAIVAFTRGLSLQLVSKGIRVNGVAPGPIWTPLIPASFK

EEETAQFGGQVPMKRAGQPIEVAPSYVFLASNQCSSYFTGQVLHPNGGTVVNG*

>MtSDR57C2

MTSNEAKFPPQTQQTQPGKEHVMEPLPQTINPDHNPTNKLRGKVALVTGGDSGIGRAVCL

IFAKEGATVAFTYVKGVEDRDKDDTLKMLLEAKTSDAQEPLAIAADIGYDENCKQVVELV

VKEYGSSIDVLVNNAAEQHLRNSIEEITEQQLERVFRTNIFSHFFLVRHALKHMKEGSSI

INSTSVNAYTGKAETLDYTSTKGAIVAFTRGLAQQLVSKGIRVNAVAPGPIWTPVQPATM

PYEKIQNLGSDVPMKRAGQPCEIAPCYLFLASLQDSSYFTGQVLHPNGGVIVNA*

>MtSDR57C3

MTTGGQKIPPQKQDTQPGKEHAMNPTPQFTCPDYKPANKLQGKIAVVTGGDSGIGRAVCN

LFALEGATVIFTYVKGHEDKDARDTLDMLKMAKTANAKDPMAIPADLGFDENCKRVIDEI
INAYGRIDILVNNAAEQYECGSVEEIDEPRLERVFRTNIFSYFFMTRHALKHMKEGSNII
NTTSVNAYKGNSTLIDYTSTKGAIVAFTRALSLQLVSKGIRVNGVAPGPIWTPLIPASFN
EEKTAQFGSDVPMKRAGQPVEVAPSFVFLASNQCSSYITGQVLHPNGGTVVNA*
>MtSDR65C1
MAKQENSSRSRSSRWSLKGFTALVTGGTRGIGHAIVEELAEFGAIVYTCSRNEEELNKCL
NEWKEKGFSVYGSVCDVTSSSQREELVRKVASAFNGKLNILVNNVGTNVRKPTIEYTTED
YSKLMTINLDSAYHLCQLTYPLLKESGNGSIVFNSSVASLTHVGSGTIYAACKAAINQLN
KGLACEWAKDNIRINCVAPWYTKTPLNDYLYANKEFVNEVLSRTPIKRIAETHEVSSLVA
FLCLPAASYITGQVISVDGGFTANGFQPSMIIRSD*
>MtSDR65C2
MENQESSMRGSRWSLKGTTALVTGGTRGIGHAVVEELAEFGATVFTCSRNEEELNKCLNE
WKEKGFSVYGSVCDASSPSQREELIRQVASAFNGKLNILVNNAGTNVRKPTIEYTAEDYS
KVMTTNLDSAYHLCQLAYPLLKESGNGSIVFISSVASLISVGTGSIYAVSKAAINQLTKN
LACEWAKDNIRSNCVAPWYTKTSLVEQFIANKEFVDEVLSRTPIKRIAETHEVSALVTFL
CLPAASYITGQTVSVDGGFTVNGFSGLFK*
>MtSDR65C3
MANAESSSRSSRWSLKGFTALVTGGTRGIGHAIVEELAEFGATVYTCSRNQEELNKRLNE
WKEKGFSVYGSVCDASSSSQREELIQNVASSFNGKLNIFVNNAGTNVRKPTIEYTAEDYS
KVMTTNLDSAYHLCQLTYPLLKESGNGSIVFISSVGSLTSVGTGSIYAASKAAINQLTKS
LACEWAKDNIRSNCVAPWYTKTPLVEHLIANEEFVNQVLSRTPIKRIAETHEVSSLVTFL
CLPAASYITGQIVSVDGGFTVNGFQPSMRIT*
>MtSDR65C4
MAAAAVETPSSRGGQRWSLTGMTALVTGGTRGIGHAIVNDLVAFGAAVHTCSRTESELSK
CLQEWQSKGFSVTGSVCDVSSRSQRESLVQEVASTFNGKLNIFVNNVGSNFRKPTIEYTA
EEYSELMTINLDSSFHLCQLSHPLLKESGNGSIVFISSVAGVVSLGTGSVYAASKAAINQ
LTKNLACEWAIDNIRSNCVVPWATRTPLVEHLFQNQKFVDDILSRTPLKRIAEPEEVSSL
VTFLCLPAASYITGQVICVDGGLTVFGFQPSMRIT*

>MtSDR65C5

MAAVEECSSNGVSRWSLKGKTALVTGGTRGIGHAIVEDLCGFGATVHTCSRNQDELNNCL

NQWRSKGFLVSGSVCDVSSREQREKLIQEVASIFNGKLHIYVNNVGANFRKPTIEYTAEV

YSEIMAINLDSAYHLCQLTHPLLKAYGMGSIVFISSIAGVVSLGTGSVYAASKAAITQLT

KNLACEWAKDGIRSNCVVPATTNTPLVEHLLRNKQYMDEMLSRTPLGCIAESHEVSSLVA

FLCLPAASYITGQVICVDGGLTVNGFQPSMRIT*

>MtSDR65C6

MLHSHTPTNHKWSLNGMTALVTGGTRGIGYAIVEELMGFGAKVHTCARNEDDLNKCLKDW

NHLGFEVTGSICDVSVPQQREVLMEDVSSVFNGKLNILINNVGTNIRKPMIDFTAAEFSR

LIDTNLGSTFHMCQLAYPLLKASGVGSVVFISSVSGFVSLKSMSVQGATKGAINQLTRNL

ACEWAKDNIRSNAVAPWYIRTSMVEQVLSNKDYLEEVYSRTPLRRLGDPAEVSSLVAFLC

LPASSYITGQIICVDGGMSINGFSPTHI*

>MtSDR65C7

MAEGNTSSRSSKWSLKGTTALVTGGSKGIGYDIVEQLAELGATVHTCARNEAELNECLNQ

WVTKGYKITGSVCDVASRAQREDLIARVSSEFNGKLNILVNNVGTNMQKQTLDFTEQDFS

FLVNTNLESAFHISQLAHPLLKASNNASIVFMSSIGGVASLNIGTIYSAAKGAIIQLTKN

LACEWAKDNIRTNCVAPGPIRTPLAAEHLKDEKLLDAFIERTPLGRIGEPEEVSSLVAFL

CLPAASFITGQTICIDGGLTVNCLLLQ*

>MtSDR65C8

MGETKLSSFKDKRWSLHGMTALVTGGTRGIGYAIVEELAEFGASVHICARNEEDINKCLE

EWKNKGFNVTGSVCDILFHEQRKKLMETVSSIFQGKLNILVNNAAKPTSKKIIDNTDEDI

NTTLGTNFVSGYHLCQLAHPLLKQSGYGSIVFTSSVAGLKAIPVLSVYAATKGAVNQFTK

NLALEWAKDNIRANAVAPGPVKTSLLESVMDYDSEGYKAIAGIVSQTPTGRMGETKEISA

LVAFLCLPAASHITGQIIAIDGGYTS*

>MtSDR65C9

MGETKLSSFKDKRWSLHGMTALVTGGTRGIGYAIVEELAEFGAAVHVCARNEEDINKCLE

EWKNKGFNVTGSVCDILFHEQRKRLMETVSSIFHGKLNILVNNAAKPTSKKIIDNTDEDI

NTTLGTNFVSGYHLCQLAHPLLKQSGYGSIVFTSSVAGLKAIPVLSVYAATKGAVNQFTK

NLALEWAKDNIRANAVAPGPVKTSLLQSITNDNEGDEAVDGVVSQTPMGRMGEPKEISSL

VAFLCLPAASYITGQVIAIDGGFTS*

>MtSDR65C10

MDETKLKFKDQRWSLQGKTALVTGGTRGIGHAIVEELVEFGARVHICARNQDDINKCLEE

WKGKGFCVTGSACDLLSCDQRQNLMENVASIFQGKLNILVNNAATCTHKKILEYTAEDIV

TTMGTNFESGYHLCQLAYPLLKESGYGSIVFISSVGGLKAFDACSIYAASKGAINQLTKN

IALEWAKDNIRANVVAPGPVNTDLLESILKSKPFEKITMDDIASSAPVGRVGEPKDISAL

VAFLCLPAASYVTGQIIAADGGFIL*

>MtSDR65C11

MVDTKLNFKDQKWSLHGMTALVTGGTRGIGYAIVEELAEFGASVHICARNQDDINKCLEE

WKGKGFCVTGSTCDLLFHDQRQKLMETVASIFDGKLNILVNNAGTITPKTMLEHTAEDVT

NTMGINFESSYHLCQLAHPLLKESGYGSIVSISSILGLRPLPLCSIYAASKGAINQCTKN

IALEYGKDNIRANVVAPGAVMTTLLESILEHPDAPKVMEVALSQTPINRVAQPRDISALV

AFLCLPAASYITGQIIAADGGFTS*

>MtSDR65C12

MAETKLSSFKDQRWSLQGMTALVTGGTRGIGYAIVEELAEFGASVHICARNQEDINKCLE

EWKGKGFCVTGSTCDLLFHDQRQKLMETVALVFDGKLNILVNNAGIFTPKPIMDYTDEDI

TTTIGTNFVSGYHLCQLAHPLLKQSGNGSIVFISSIAGLKAWPFASVYAASKGAMNQCAK

NLALEWAKDNIRANVVAPGAVMTILFENAAKLAGRVDNVVEDMVSLTPSGRIGEPKDISG

LVAFLCLPAASHITGQIIAADGGFTI*

>MtSDR65C13

MAETKLSSFKDQRWSLQGMTALVTGGTRGLGYAIVEELAEFGASVHICARNQDDINKCLE

EWKGKGFCVTGSTCDLLFREQREKLMETVASTFQGKLNILVNNAGIFTPKPIMDYTDEDI

ASTIGTNFVSSYHLCQLAHPLLKQSGYGSIVYISSISGLKALPFVSVYAASKGAMNQCTK

NLALEWAKDNIRANVVAPGPVMTLLLENAMKLVAGVDNAIKDIVSQTPGGRIGEPKDISG

LVAFLCLPAASHITGQIIAADGGFTI*

>MtSDR67E1

MSSSSSTRLDLDGNSIKPITICIIGAGGFIGSHLCEKLMLQTPHKVIALDVYKNKLKHLL

EPETLPWNNRFEFHRLNVKNDSRLEGLIKTADLVINLAAICTPADYNTRPLDTIYSNFID

AIPVVKYCSENNKRLVHFSTCEVYGKTIGSFLPKDSPLRQDPAYYMLKEDESPCIFGPIE

KQRWSYACAKQLIERLIYGEGDENGLEFTIVRPFNWIGPRMDFIPSVDGPSEGVPRVLAC

FSNNLLRGEPLKLVDGGESQRTYLYIKDAIEAVLLMIENPARANGKIFNVGNPNNEVTVR

ELAEMMIKVYSKVSGDQPLETPTVDVSSKEFYGEGYDDSDKRIPDMTIINKQLGWNPKTS

LEDLLESTLTYQHKDIC*

>MtSDR67E2

MCSPSSTRLNLDGNPIKPITICIIGAGGFIGSHLCEKLMLQTPHKVLALDVYSDKLKHLL

EPDTLPWNNRIDYHSLNIKNDSRLEGLIKIADLVINLAAICTPADYNTRPLDTIYSNFVD

AIPVVYGKTIGSFLSKDSPLRHDPAYYMLKEDESPCIFGPIEKQRWSYACAKQLIERLIY

GEGDEKGLEFTIVRPFNWIDPRMDFIPGVDGPSEGVPRVLACFSNNLLRGEPLKLVDGGE

SQRTFLYIKDAIEAVLLMIENPARANGKIFNVGNPNNEVTVRELAEMMIKVYSKVSGDQP

LETPTIDVSSKEFYGEGYDDSDKRIPDMTIINKQLGWNPKTSLEDLLESTLTYQHKTYAE

AIKKVIAQTIAS*

>MtSDR67E3

MSSPSSIRLDLDGNPIKPITICIIDTGGFIGSHLCEKLMLQTPHKVLALDVYNNKLKHLL

ELETLPWNNHFEFHRLNIKNDPILESLIKTTYLVINLVAIYTPIDYNTSPLHTIYNNFIV

AIPVVKHCSENNKRLVHFSTCEVHGKTIGSFLPKDSPLRHDPAYYMLKEDESSCIFGDEN

GLEFTIVRPFNWIGPRMDLVPSVDGPSERVPRVLACFSNNLLRGEPLK*

>MtSDR67E4

MDRVNLDGKPIVPISICLIGGGGFIGSHLTEKLMSETSHKAIVIDVSSEKVNHLLDKSHP

WANRIEFHQMNIKNDSRLETLVKASDLTINLAAICTPADYNTRPLDTIYSNFIDAIPVIK

FCTENNKRLIHFSTCEVFGKTIGSFLPEEYRKEPQYYKLKEDVSPCIFGPVHKQRWSYAC

AKQMTDRLIYAEHAENGLKFTIVRPYNWIGPRMDFIPGVDGPSDGVPRVLACFSNNLLRG

EPLKLVDGGHSQRTFLYIKDAIEAVMLMIDNPDRANGHIFNVGNPDNEVSVKQLAELMIK

VYAKVAGVPESSLSTLDVSSEVFYGKGYDDMtSDRRIPDMTIITKQLGWKPKTSLDDLLDST

LQYQHQTYSHAIKKELSKPST*

>MtSDR67E5

MASPSSTRIDLDGNPIKPLTICMIGAGGFIGSHLCEKLMSETSHKVLALDVYNDKIKHLL

EPESLPWNGRIHFHRLNIKNDSRLEGLIKMADLVINLAAICTPADYNTRPLDTIYSNFID

ALPVVKYCSETNKRLIHFSTCEVYGKTIGSYLPKDSPLRQDPAYYMLKEDVSPCIFGSIE

KQRWSYACAKQLIERLVYAEGAENGLEFTIVRPFNWIGPRMDFIPGIDGPSEGVPRVLAC

FSNNLLRGEPLKLVDGGESQRTFVYIKDAIEAVLLMIENPARANGHIFNVGNPNNEVTVR

QLAEMMIQVYSKVSGTQPPETPTIDVSSKEFYGEGYDDSDKRIPDMTIINKQLEWNPKTS

LWDLLESTLTYQHRTYAEAIKKVIAQPIAS*

>MtSDR68C1

MATTPQQVIEPLPLQNRVAIITGSSRGIGREIAIHLSSLGARIVINHSSSNSLSADSLAA

DINATSPLPRATVIGADISDQSQVQSLFDSAERFFNSPIHILINCAGVIDDTYPSIANTS

IESFDRVFGVNARGAFLCAREAANRLKRGGGGRIILFSSSQVAALRPGFAAYTAAKAAVE

TMTKILAKELKGTGITANCVAPGPIATEMFFGGRTEEQVQKIIDESPLGRLGETKDVAPL

VGFLASDAGEWVNGQIIRINGDDMADGATFAVVDLSKTFALPL*

>MtSDR68C2

MASLPLQDRVAIVTGSSRGIGKEIALHLASLGARLVINYTSNSNNADSVAAEINANQTTF

RAITVRADVSDPEGVKSLFDSAEEAFKSPVHILVNSAGVLDAKYPTIANTTVESFDRIMN

INLKGAFLCTKEAANRLKRGGGGRIILLTSSLVAALKICMGAYTASKAAVEAMTKILAKE

LKGTGITANCVAPGPIATEMFFEGKTEEMVKKTEEESPFGRLGETKDVAPVVGFLATDAS

EWVNGQIIRVNGGYV*

>MtSDR68C3

MATSLPQSPPLQDRVAIVTGSSRGIGKEIALHLASLGARLVINYTSNSHLADSVAAEINA

NQTTPRAITVRADVSDPEGVKSLFDSAEQAFNSPVHILVNSAGVLDAELPTIANTTVETF

DRIMNVNARGAFLCAKEAANRLKRGGGGRIIFLTTSLAAAFKPGYGAYTASKAGVEAMTK

ILAKELKGTGITANCVAPGPTATELFLEGKSEERVKMIAESNPFGRIGETKDISPVVGFL

ASDSGEWVNAQIIRVNGGFV*

>MtSDR73C1

MALQATSCLPASFSITKEGKIGASLKDSTFFGVSLSDSLKGDFSSSALRCKRELRQKVGA

VRAETAATATPAVTKSSPDGKKTLRKGSVVITGASSGLGLATAKALAETGKWHVIMACRD

FLKASRAAKSAGMAKENYTIMHLDLSSLDSVRQFVDNFRRSEMPLDVLVNNAAIYLPTAK

EPTFTAEGFELSVGTNHLGHFLLSRLLLEDLGKSDYPSKRLIIVGSITGNTNTLAGNVPP

KANLGDLRGLAGGLNGLNSSAMIDGGDFDGAKAYKDSKVCNMLTMQEFHRRYHEETGITF

ASLYPGCIATTGLFREHIPLFRTLFPPFQKYITKGYVSEDEAGKRLAQVVSDPSLTKSGV

YWSWNKASASFENQLSQEASDVEKARKVWEVSEKLVGLA*

>MtSDR75U1

MNWWLAKASGTGKASYLSIITEFKEAPGSFQNVALIIGVTGIVGNSLAEILPLDDTPGGP

WKVYGVARRPQPTWNADKYVHYIQCDVSDQKDVELKLSPLTDVTHIFYVSWTSMPTEAQN

CEVNGSMLRNVLRALIPNTLNLCHVSLQTGTKHYLGSFETIGKIKAHESPFTEDVPRLVT

PNFYYTQEDILLEEVGKKKGTTWFINRPHPIFGFSPYSMMNVIGTLCVYAAICKHEGLPL

RFPGSKGAWECYSTASDANLIAEQHIWGAADPNAKNEAFNCSNGDVFKWKHLWKVLAERF

EIDDYGFEEGSELRLSDLMKDKGGVWEEIVRENELLYTKLEEVGDWWFADFSLRLEGVLD

SMNKAKEHGFIGFRNSKNSFISWIDKTKAYKIVP*

>MtSDR81U1

MKMATLMRLPTATPGRVVTRTREAFHALSPSPSPSPPHFVEFCKGGRRGRRIIRVGVKCN

SNSERAVNLGPGTPVRPTSILVVGATGTLGRQIVRRALDEGYDVRCLVRPRPAPADFLRD

WGATVVNADLSKPETIPATLVGVHTVIDCATGRPEEPIKTVDWEGKVALIQCAKAMGIQK

YVFYSIHNCDKHPEVPLMEIKYCTENFLRDSGLNHIVIRLCGFMQGLIGQYAVPILEEKS

VWGTDAPTRIAYMDTQDIARLTFIALRNEKINGKLLTFAGPRAWTTQEVITLCERLAGQD

ANVTTVPVSVLRLTRQLTRFFEWTNDVADRLAFSEVLTSDTVFSVPMAETYNLLGVDTKD

IITLEKYLQDYFTNILKKLKDLKAQSKQSDIFF*

>MtSDR83U1

MAMLASSSPTLLFTSTSSNLLPLSHSCTLRLSFSSSLQSTSLSISSTFLSHPSLTSKRLA

NHATLSISASAAEKKKVLIINTNSGGHAVIGFYFAKELLGAGHSVTILTVGDESSDKMKK

PPFNRFSEIVSAGGSTVWGNPADVGSVVGGEAFDVVLDNNGKDLESVRPVIDWAKSAGAK

QFLFISSAGIYKTTDEPPHVEGDAVKADAGHVGVEKYIEETFDSWAVFRPQYMTGSGNNK

DCEEWFFDRIVRDRPVLIPGSGLQLTNISHVKDLSSMLTLAVENPDAANHSIFNCVMtSDRA

VTLNGIAKLCAQAAGRPVNIVHYDPKAIGIDAKKAFPFRNVHFYAEPRAAKTKLGWSSTT

NLPEDLKERFEEYIKIGRDKKPIKFELDDKILEALKVPVSV*

>MtSDR83U2

MARLVALQQNQLSFPPLASSLSDFNGARLQTHIQLKRKTWQPKGSLIVSASSTKKILIMG

GTRFIGVFLSRQLVKEGHQVTLFTRGKAPITQQLPGESDTDFADFSSKILHLKGDRKDYD

FVKSSLSAEGFDVVYDINGREAEEVEPILDALPNLEQFIYCSSAGVYLKSDLLPHAEIDA

VDPKSRHKGKLETESLLQSKDVNWTSIRPVYIYGPLNYNPVEEWFFHRLKAGRPIPIPGS

GIQITQLGHVKDLATAFLKVLGNEKASKQIFNISGDKYVTFDGLARACAKAGGFPEPEII

HYNPKDFDFGKKKSFPFRDQHFFASVEKAKSVLGLEPDYGLVEGLTDSYNLDFGRGTFRK

EADFSTDDIILGKSLVSV*

>MtSDR84C1

MASSRGNIAAIVGVGPNLGLSIARKFAHEGYTVAILARDLGRLSRFADEIAREEKAQVFA

IRIDCSDSRSVREAFEGVLSLGFVEVLVYNANYESPLQSKPTPFQHLPIQTFQTSLAVSS

LGAFNCAQQVLPGMVERGKGTILFTGCSASLNGIAGYSELCCGKFALRALSQCLAREFQP

QGVHVAHIIIDGFIGPPRGSSATTTSSRGGGSGGESVMDPDALAQTYWHIHVQDRNAWTQ

EMDVRSSNF*

>MtSDR87D1

MATTTVSNMSIAMSRPRITPSQKFSNLGPAIVGSRVKVGSCAKLGSVCHVALAKPFQLSS

TSHTLKFDKIVTKAMAESSSNKQATGLPIDLRGKRAFIAGVADDNGYGWAIAKSLAEAGA

EILVGTWVPALNIFESSLRRGKFDESRKLQDGSLMEITKVYPLDAVYDTPEDVPEDVKAN

KRYAGASNWTVQEVVESVKKDFGTIDILVHSLANGPEVSKLLSETSRKGYLAALSASSYS

YISLLKHFLPIMNQGGSSLSLTYIASERIIPGYGGGMSSAKAALESDTRVLAFEAGRKKR

IRVNTISAGPLGSRAAKAIGFIDMMIDYSFTNAPLQKELHAEEVGNAAAFLASPLASAIT

GAVIYVDNGLNAMGVGVDSPVFKDLDIPRDQH*

>MtSDR93E1

MGSTEKTNYGEYTYENLEREPYWPSEKLKISITGAGGFIASHLARRLKKEGHYIIASDWK

KNEHMTEDMFCDEFHLVDLRVMDNCLTVTKGVDHVFNLAADMGGMGFIQSNHSVIMYNNT

MISFNMIEAARINGIKRFFYASSACIYPEFKQLETTNVSLKESDAWPAEPQDAYGLEKLA

TEEICKHYNKDFGIECRIGRFHNIYGPFGTWKGGREKAPAAFCRKAITSTDKFEMWGDGL

QTRSFTFIDECVEGVLRLTKSDFREPVNIGSDEMVSMNEMAEIVLGFEDKKTPIHHIPGP

EGVRGRNSDNTLIKEKLGWAPTMKLKDGLRITYVWIKEQLEKEKAQGLDTSGYGSSKVVS

TQAPVQLGSLRAADGKEGSS*

>MtSDR93E2

MSFGLHLIVINFDVIFYSKTQSNLNSSFTPLFIRMGSSGINNDYGAFTYQNLEREPYWPT

EKLRISITGAGGFIASHIARRLKTEGHYIIASDWKKNEHMTEDMFCHEFHLVDLRVMDNC

LKVTKDVDHVFNLAADMGGMGFIQSNHSVIMYNNTMISFNMIEAARINGVKRFFYASSAC

IYPEFKQLETNVSLKEADAWPAEPQDAYGLEKLATEELCKHYNKDFGIECRIGRFHNIYG

PFGTWKGGREKAPAAFCRKTLTSTDKFEMWGDGLQTRSFTFIDECVEGVLRLTKSDFREP

VNIGSDEMVSMNEMAEIVLSFENKSIPIQHIPGPEGVRGRNSDNTLIKEKLGWAPTMKLK

DGLRITYFWIKEQLEKEKAGGVDVTSYGSSKVVSTQAPVQLGSLRAADGNE*

>MtSDR98U1

MSLCYSSSTFITHPSLKHQTLSFNFSSHPPSHFINLFKVKSNRPIKYTKQKLKLYASLSQ

SEQIETTPTTFRIKNPKDVNVLVVGSTGYIGKFVVKELIQRGFNVTAIAREKSGIKGSID

KETTLNELRGANVCFSDVTNLDVFDEDLKNLGVGFDVVVSCLASRNGGVKDSWKIDYEAT

KNSLLAGRKLGASHFVLLSAICVQKPLLEFQRAKLKLEDELVKEAEKDDRFSYSIVRPTA

FFKSLGGQVDLVKDGKPYVMFGDGKLCACKPISEQDLASFIVDCVMSEDKINKILPIGGP

GKALTPLEQGEILFKLLRKEPKFLKVPIGIMDFAIGVLDNLVKVFPSLEDAAEFGKIGRY

YAAESMLILDPDTGEYSDEKTPSYGNDTLEDFFARVLREGMAGQELGEQTIF*

>MtSDR108E1

MGSVSETVCVTGASGFIGSWLVMRLMERGYTVRATVRDPDNMKKVKHLLELPGANSKLSL

WKADLGEEGSFDEAIKGCTGVFHVATPMDFESKDPEKEVINPTINGLLDIMKACKKAKTV

RRLVFTSSAGTLDVTEQQNSVIDETCWSDVEFCRRVKMTGWMYFVSKTLAEQEAWKFSKE

HNIDFVSIIPPLVVGPFIMPSMPPSLITALSLITGYEAHYSIIKQGQYIHLDDLCLAHIF

LFENPKAHGRYICCSHEATIHEVAKLINKKYPEFNVPTKFKDIPDDLEIIKFSSKKITDL

GFIFKYSLEDMFTGAIETCREKGLLPKVTETPVNDTMKK*

>MtSDR108E2

MGSMAETVCVTGASGFIGSWLVMRLMERGYMVRATVRDPENLKKVSHLLELPGAKGKLSL

WKADLGEEGSFDEAIKGCTGVFHVATPMDFESKDPENEMIKPTIKGVLDIMKACLKAKTV

RRFIFTSSAGTLNVTEDQKPLWDESCWSDVEFCRRVKMTGWMYFVSKTLAEQEAWKFAKE

HNMDFITIIPPLVVGPFLIPTMPPSLITALSPITGNEAHYSIIKQGQFVHLDDLCEAHIF

LFEHMEVEGRYLCSACEANIHDIAKLINTKYPEYNIPTKFNNIPDELELVRFSSKKIKDL

GFEFKYSLEDMYTEAIDTCIEKGLLPKFVKSTNK*

>MtSDR108E3

MASENREEVVCVTGANGFIGSWLVHTLLSKQNPHYKIHATIFPNSDPSHLFTLHPEAQSR

ITIFPVNILDSTAVSNAINGCSGVFHVASPCTLEDPTDPQKELLEPAVQGTLNVLEASKR

AGVKRVVLTSSISAMVPNPNWPENKAIDEGSWTDVEYCKLRGKWYPVSKTEAEKVAWEFC

EKHSGVDVVAVHPGTCLGPLLQNQMNASSAVLQRLMMGEKDTQECYWLGAVHVKDVARAH

VLVYETPTAAGRYLCVNGIYQFSSFAKIVSELYHDYPIHSFPNETQPGLTPFKEAAKRLI

DLGLVFTPIQDAIREAAESLMAKGFLQRTTSQK*

>MtSDR108E4

MEHKVCVTGASGFLASWLIKRLLLSGYHVIGTVRDLGKKQKVEHLWKLEGATERLKLVQA

DLMEENSFDNAIMGCKGVFHIASPVLNHISNDPKAEILEPAVQGTLNVLRSCRKNPALVR

VVLASSSSAVRVRADFDPNIPIDESSWSSLELCEKLQAWYPMSKTLAEKAAWDYCKENGI

DLVTILPSFIIGPNLPTDLCSTASDVLGLFKGETEKFQWHGRMGYVHIDDVALCHILLYE

NKASDGRYLCSSKIMDNDDLVGMLANRYPGFPIPKRFKKLDRPHYELNTGKLESLGFKFK

SVEEMFDDCFASFVEQGHLTTLPHQPRVIL*

>MtSDR108E5

MPAATAAAAAESSSVSGETICVTGAGGFIASWMVKLLLEKGYTVRGTLRNPDDPKNGHLK

KLEGAKERLTLVKVDLLDLNSVKEAVNGCHGVFHTASPVTDNPEEMVEPAVNGAKNVIIA

GAEAKVRRVVFTSSIGAVYMDPNRSVDVEVDESCWSDLEFCKKTKNWYCYGKAVAEAAAW

DVAKEKGVDLVVVNPVLVLGPLLQPTINASTIHILKYLTGSAKTYANATQAYVHVRDVAL

AHILVYEKPSASGRYLCAETSLHRGELVEILAKYFPEYPIPTKCSDEKNPRVKPHIFSNK

KLKDLGLEFTPVSECLYETVKSLQDQGHLSIPNKEDSLAVKS*

>MtSDR108E6

MSSSNLGNVVCVTGASGYIASWLVRLLLHRGYTVKATVRDPNDPKKVDHLVKLDGAKERL

QLFKANLLEEGAFDSVVQGCHGVFHTASPFYHDVKDPQAELIDPALKGTLNVLNSCAKSP

SLKRVVLTSSIAAVAYNGKPRTPDVVVDETWFTDADFCAKSNLWYVVSKTLAEEAAWKFV

KENNIDMVTINPAMVIGPLLQPVLNTSAAAILNLINGAQTFPNASFGWVNVKDVANAHIL

AYENASASGRHCLVERVAHYSEVVRILRELYPSLQLPEKCADDKPYVPIYQVSKEKAKSL

GLEYTPLEVSIKETVESLKEKKFANL*

>MtSDR108E7

MNSNVVCVTGASGYIASWLVRYLLHRGYTVKATVRDPSDPKKINHLVKLEGAKERLQLFK

ANLLEQGAFDSAVQGCHGVFHTASPFYHHVKDPQAELIDPALNGTLNVLKSCAKSPLLKR

VVLTSSAAAVAYNEKPRTPDVVVDETWFTDADFCAKLNLWYAVSKTLAEEAAWKFVKENN

IDMVTINPAMVIGPLLQPVLNTSAAAILNLINGTQTFPNSTLGWVYVKDVANAHILAYEN

ASASGRHCLVESVAHYSEIVKILRELYPSLQLPEKCADDKPYVPTYQFSKEKATSLGIEY

TPLEVSIKETVESLKEKKFVNF*

>MtSDR108E8

MSKTVCVTGASGAIGSWVVRLLLERGYTVHATIQDLEDENETKHLEAMEGAKTRLKFFEM

DLLNSDSIAAAVKGCAGVIHLACPNIIGEVKDPEKQILEPAIQGTVNVLKVAKEAGVERV

VATSSISAIIPSPSWPADKIKAEDCWTDLEYCKEKKLYYPIAKTLAEKAGWEFAKETGFD

VVMINPGTALGPLIPPRINSSMAVLAGVLKGDKETYEDFFMGMAHFKDIALAHILGFEQK

KASGRHLCVEAIRHYSDFVNLVAELYPEYNVAKIPTDTQPGLLRAKNASKKLIELGLEFT

PAEEIIKDAVECLKSRGLV*

>MtSDR108E9

MPAYDNTSSVSGGDQTVCVTGAGGFIASWLVKLLLERGYTVRGTVRNPEDPKNGHLKELE

GARERLTLHKVDLLDLQSIQSVVHGCHGVFHTASPVTDNPDEMLEPAVNGTKNVIIASAE

AKVRRVVFTSSIGTVYMDPNTSRDVVVDESYWSDLEHCKNTKNWYCYGKTVAEQSAWDIA

KENQVDLVVVNPVVVLGPLLQPTINASTIHILKYLNGAAKTYVNATQSYVHVKDVALAHL

LVYETNSASGRYICCETALHRGEVVEILAKYFPEYPLPTKCSDEKNPRVKPYKFSNQKLK

DLGLEFTPVKQCLYDTVRSLQEKGHLPIPPMQEDSA*

>MtSDR108E10

MEASGGVNNNKKVCVTGAGGFVASWLVKLLLSKGYFVHGTVREPGSPKYEHLLKLEKASE

NLTLFKADILDYESVYSAIVGCSAVFHVASPVPSTVVPNPEVEVIEPAVKGTANVLEACL

KANVERVVFVSSAAAVAINPNLPKDKAIDESCWSDKDYCKNTQNWYCYAKTEAEEQAFYF

AKRTGLNVVTICPTLVLGPILQSTTNSSSLALIKILKEGHDSLENKLRWIVDVRDVVNAI

LLAYENHKADGRYICTSHTINTRDLVERLKSIYPNYKYPTNYIEMDDYKMLSSEKLQSLG

WKFRPLEETLIDSVESYKEAGLLQSA*

>MtSDR108E11

MEASGGVNNNKKVCVTGAGGFVASWLVKLLLSKGYFVHGTVREPGSPKYEHLLKLEKASE

NLTLFKADILDYESVYSAIVGCSAVFHVASPVPSTVVPNPEVEVIEPAVKGTANVLEACL

KANVERVVFVSSVAAVAINPNLPKDKAIDESCWSDKDYCKNTKNWYCYAKTEAEEQALHF

AKRTGLNVVTICPTLVLGPILQSTTNASSLVLVKLLKEGCDSVENKLRWIVDVRDVVNAI

LLAYENHEADGRYICTSHAIVTRDLVERLKGIYPNYKYPTNYIEMDDYKMLSSEKLQSLG

WKLRPLEETLIDSVESYKEAGLLQSQ*

>MtSDR108E12

MENDKGTICVTGATGYVASWLIMKLLQHGYAVHATVRSHHVKEKKDLSYLTNLPEASKKL

TIFHADLDDSSSFEKAIQGCIGVFHLAHPMDVQNQEPEEKVTKRAVEGTLEILKACLESK

TVKKVVYTSSAATVLFNDKNLDVVDEDIWSDIDICRSSNLVGSSYLVSKIMTEKSVLEFG

KVNELEVVSLVLPLVVGPFICPNIPSSVYIALAMIFGDQDRYKYLTNSYMVHTDDATRAL

IFLFESENVNGRLICSMtSDRISFHQLYELLCQRYPGYNITIPNSMNTKNGDKKFSDLSSKK

LLDTGFKFKYSVNDMYDGAIQCCKEKGIL*

>MtSDR108E13

MTMKSGEGKVVCVTGASGFIASWVVKFLLQRGYTVRATVRDPSNSNKVDHLLKLDGAKER

LQLFKADLLEEGSFDSVIQGCHGVFHTASPVHFVVTDPQTQLIDPAVKGTLNVVKSCAKS

PSVQRVVLTSSIATALYTGKPRTPEVEVDETWFSNQDFLWQNKMWYQFAKTSAEEAATKF

LTENNIDHVVMNPAVALGPLLQSELNESSTLILNLINGSETYMNAAFGWINVKDIANAHI

QAYENASASGRYCLVERVIHFSELAKILRDMYPTLQIPDKCADDKPLMQTFQVSKEKAKT

LGVEFIPLEVSLREIVESFKDKKFANF*

>MtSDR108E14

MNSSEGKVVCVTGASGYIASWLVKFLLQRGYTVRATVRDPSNPNKVDHLLKLDGAKERLH

LFKADLLEEGSFDHVIQGCHGVFHTASPAHFVVDDPQTQLIDPAVKGSLNVLRSCAKSPS

VKRVVFTSSIATALYNGTPRTPDTVVDETWFSNLDLLREQKMWYQFAKVSAEEAATKFLT

ENDIDYVVMNPAVTIGPLLQPELNGSSSLIFDLIKGSQTFLNATFGWINVKDVANAHIHA

YEDASTSGRYCLAERVAHISELAIILRDMYPTLQIPDKCAGDKPLMQTFQISKEKAKTLG

IEFIPLEVSLREIVESFKEKEFANF*

>MtSDR108E15

MKEEMEEAMRMVRSSGQIVPTAKYCVTGATGYIGSWLVEALLQRGCTVHATVRDPEKSLH

LLSLWKGGDQLRFFRADLHEEGSFEEAVKGCDGVFHVAASMQFNVNEKENIENFVQTNII

DPAIEGTVNLLKSCLKSNSVKRVVFTSSISTITAKDSNGKWKPIVDESCQIQTDTVWNTQ

PSGWVYALSKLLTEEAAFKFAKENGIDLVSVITSTVAGPFFTANVPSSVKVLLSSLTGET

ENYKILSAVNARMGSIALVHIEDICNAHIFLMEHAKAEGRYICSTQSCTLSNLAALVSKE

YSNPTTQSRKSQKYDKVPTEISSKKIKDLGFSYKHSLEEIVHQTIMCCLDYGYLPSV*

>MtSDR108E16

MASIKQIEIEKKKACVIGGTGFVASLLIKQLLEKGYAVNTTVRDLDSANKTSHLIALQSL

GELNLFKAELTIEEDFDAPISGCELVFQLATPVNFASQDPENDMIKPAIKGVLNVLKACV

RAKEVKRVILTSSAAAVTINELEGTGHVMDETNWSDVEFLNTAKPPTWGYPVSKVLAEKA

AWKFAEENNIDLITVIPTLTIGPSLTQDIPSSVAMGMSLLTGNDFLINALKGMQFLSGSI

SITHVEDICRAHIFVAEKESTSGRYICCAHNTSVPELAKFLSKRYPQYKVPTEFDDFPSK

AKLIISSGKLIKEGFSFKHSIAETFDQTVEYLKTQGIK*

>MtSDR108E17

MPEFCVTGGTGFIAAYLVKALLEKGHIVRTTVRNPDDLEKVGYLTQLSGDKERLKILKAD

LMVEGSFDEAVTGVDGVFHTASPVIVPYDNNIQATLIDPCIKGTQNVLNSCIKANVKRVV

LTSSCSSIRYRDDVQQVSPLNESHWSDPEYCKRYNLWYAYAKTLGEREAWRIAEESGLDL

VVVNPSFVVGPLLAPQPASTLLMILSIVKGSRGEYPNTTVGFVHIDDVIAAHILAMEEPK

ASGRLVCSSTVAHWSQIIQMLQAKYPSYPYETKCSSQEGDNNTHSMDTTKITQLGFSQFK

SLEQMFDDCIKSFQDKGFL*

>MtSDR108E18

MSKVVCVTGGSGCIGSWLVHLLLHRGYIVHATVQNLNDENETKHLQALEGAQTNLRLFQI

DLLNYDTVLAAVHGCDGVFHLASPCIVDKVLDPQKELLDPAIKGTLNVLTAAKEVGVKRV

VVTSSISAIIPSPNWPSDVVKREDCWTDVEYCKQKELWYPMSKTLAEKAAWDFSKENGLD

VVVVNPGTVMGPVIPPRINASMLMLVRLLQGCTETYEDFFMGLVHFKDVALAHILVYENK

EATGRHVCVEAITHYGDFAAKVAELYPEYNVPKIQRDTQPGLLRAKDGSKKLMDLGLEFI

PMEQIIRDAVESLKSKGLIS*

>MtSDR108E19

MAEGKGRVCVTGGTGFLGSWIIKSLLENGYSVNTTIRADPERKRDVRFLTNLPGASEKLH

FFNADLSNPDSFAAAIEGCVGIFHTASPIDFAVSEPEEIVTKRTVDGALGILKACVNSKT

VKRFIYTSSGSAVSFNGKDKDVLDESDWSDVDLLRSVKPFGWNYAVSKTLAEKAVLEFGE

QNGIDVVTLILPFIVGRFVCPKLPDSIEKALVLVLGKKEQIGVTRFHMVHVDDVARAHIY

LLENSVPGGRYNCSPFIVPIEEMSQLLSAKYPEYQILTVDELKEIKGARLPDLNTKKLVD

AGFEFKYTIEDMFDDAIQCCKEKGYL*

>MtSDR108E20

MEEGKGRVCVTGGTGFIGSWIIKRLLEDGYTVNTTVRSNPGQKKDVSYLTNLPNASQNLQ

IFNADLCNPESFDAAIEGCIGIFHTATPIDFEENEREEIVTKRTIDGALGILKACKNSKT

VKRVIYTSSASAVYMQDKEEDVMDESYWSDVNILRNLKPFAWSYAVSKTLAEKAVLEFGE

QHGLDIVTLVPSFVVGPFICPKLPGSIFSSLAFLFGDIDNNPLAASRLHMVHVDDVARAH

IFLLEHPNPKGRYNCSPFLATIDEIVHIVSSKYPKFQIPTSKELMGVKGPKLPHLTSKKL

MDDGFKFKYSLEEMFEDAIECCKENGYL*

>MtSDR108E21

MEKGKGRVCVTGGTGFIGSWIIKRLLEDGYTVNTTVRSNPGQKKDVSFLTDLPNASQKLQ

IFNADLSNPESFNAAIEGCIGVFHTATPVDFELKEPEEIVIKRTIDGALGILKACKNSKT

VKRVVYTSSASAVCMQNKEVEVMDESYWSDVNNLRTLKPFAWSYAVSKTLAEKAVLEFGE

QHGLDIVTLLPTFVVGPFICPKLPSSVHSSLAFLFGGINKNPLMLVSRTGMVHVDDVARA

HIFLLEHPNPKGRYNCSPFIANIEEIVDLVSSKYPELQMPTSKELMGVKGPKFPHLTSKK

LMDDGFKFKYSLEEMFEDAIECCKEKGYL*

>MtSDR108E22

MEEGKGRVCVTGGTGFIGSWIIKRLLEDGYTVNTTVRADPGQNKDVSFLTNLPNATQKLN

IFNADLSNPKSFNAAIEGCIGVFHTATPIDFELEEPEEIVTKRTIDGALGILKACKNSKT

LKRVIYTSSASAVYTQDKEEDTMDESYWSDVNILRTLKPFAWSYGVSKTLAEKAVLEFGE

QHGLDIVTLVPPFVVGPFICPKLPGSVHSLLAFLFGDIDKHSLLLVSRTGMVHVDDVARA

HIFLLEHPNPKGRYNCSPFIATIDEIADLVSSKYPELQMPTSKELIGVKGSKLPQLTSKK

LMDAGFKFKYSLEKMFEDAVECCKEKGYL*

>MtSDR108E23

MTDRKKDVSFLTNLPGASQKLIFFGADLSIPESFNAAIEGCSGIFHTASPMDMEMNESEE

TVTKRTIDGALGILKACKNSKTVKRVIYTSSASAVYWQDKDDNVMDESYWSDENILRDLK

PFGWSYSISKTMAEKVVLEFGEQLGLDVVTIIPTLVVGSFICPKLPGSIYTSLSLLFGDK

NPFGFSHLPMVHVDDIARAHIFLLEHPNPKGRYNCSPFMANIEEIAQHISSKYPEIHIPT

LEELKDIKGDKLPHLTSKKLMDAGFEFKHSLEEMLDDTIQCCKDKAYL*

>MtSDR108E24

MEEGKGRVCVTGGTGFLGSWIIKRLLEDGYTVNATVRDDPERKKDVSFLTNLPGASQKLK

FFSADLSIPESFNAAIEGCIGIFHTATPIDLEMNESEETVTKRTIDGTLGILKACKNSKT

VKRVIYTSSASAVYWQDKDDDVMDESYWSDENLLRDLKPFAWSYSISKTMAEKAVLEFGE

QHGLDVVTIIPTFVLGPFICPKRPGSIYTSLSLLFGDNNPFGFSRLHMVHVDDIARAHIF

LLEHPNTKGRYNCSPFIANIEEIAQLISAKYPEFRIPTLEELKEIKGDKLPHLTSKKLMD

AGFEFKHSLEEMLDDTIQCCKEKGYL*

>MtSDR108E25

MTSQQEGGWMMSGEGKVVCVTGANGFIASWIVKFLLQCGYTVRATVRHPSNSEKVDHLVK

LDGAKERLQLFKADLLEEGSFDSVVEGCDGVFHTASPVRFVVNDPQAELIDPALKGTLNV

LQSCAKSPSVKRVILTSSISAVVFDTRPKNPGVIVDETWFSNPDLCRESKLWYTLSKTLA

EAAAWKFVNENSIDMVAINPTMVAGPLLQPELNGSVEPILNLISGIPFPNKAYGWCNVKD

VANAHILAYETASASGRYCLAERVVHYSELAMILRDLYPTLQISDKCEDDGPYMPTYQIS

KEKAKSLGIEFTSLEVTLKETVESFREKKIVNF*

>MtSDR108E26

MSGEGKVVCVTGASGFIASWIVKFLLQRGYTVRATVRDPSNPKKVDHLLKLDGAKERLQL

FKADLLEEGSFDSVVEGCDGVFHTASPVRFVVNDPQVELIDPALKGTLNVLKSCAKSTSV

KRVVLTSSNAAVSFNTRPKNPEVVVDETWFSNPDFCRESKLWYVLSKTLAEAAAWKFVNE

NNIDMVSLNPTMVAGPLLQPEVNESVEPILNLINGIPFPNKAIGWVNVKDVANAHIHAYE

IASASGRCLLAERVVHYSELAMILRDLYPTLPISDKCEDDGPYMPTYQISKEKAKSFGIE

FTPLEISLKETVESFREKKFIDF*

>MtSDR110C1

MSKQRLEGKVAIVTGAASGIGAEAVKIFVENGAFVVIADINDELGHQLATSIGLDKVSYH

HCDVREEKQVEEIVAFTLERYGTLDIMFSNAGNAGPLSSILEFNLNEFDNTMAVNIRGAA

ATIKHASRVMVERKIRGSIICTASVAGFVGRCAGHDYTASKHGLIGLVRSACGELGAYGI

RVNSISPYAVATPLACRALDMVPSEVEAVGLDYANLQGITLKAAHIAETALFLASDESAY

ISGHNLVVDGGFSVINRCLPSIKKQ*

>MtSDR110C2

MSTAINASTSAPAQRLLGKVAVVTGGASGIGESIVRLFHTHGAKVCIADIQDDLGQKLFD

SLSDLENVFFVHCDVAVEADVSTAVSIAVAKFGTLDIMVNNAGISGAPCPDIRNVDMAEF

DKVFNINVKGVFHGMKHAAQYLIPKKSGSIISISSVASSLGGLGPHGYTGSKHAVWGLTK

NVAAELGNHGIRVNCVSPYCVATGLALAHLPEDERTEDAMAGFRSFVGKNANLQGVELTA

DDVANAVLFLASDDAKYISGENLMVDGGFTRTNHSLKVFR*

>MtSDR110C3

MLRICLRKKKNLPYSRPLFAESINRLLSTQTGSGIGKAAATKFINNGAKVIIADIQQQLG

QETAKELGPNATFITCDVTKESDISDAVDFAVSEYKQLDIMYNNAGIPCKTPPNIVDLDL

ESFDKVMKINVRGVMAGIKHAARVMILRGTGSILCTASVTGVIGGMAQHTYSVSKFVVIG

IVKSMASELYRHGIRVNCIPPFAIPTPFVMSEMEQIYPHLDSQRLVEIVRNVGVS*

>MtSDR110C4

MLNSFTRLEGKVALITGGASGIGEATARLFSEHGAQVVIADIQDDKGHSICKELQKSSSS

YVRCDVTKEEDIENAVNTTVFKYGKLDIMFNNAGISGVNKTKILENKLSEFEDVIKVNLT

GVFLGTKHAARVMIPARRGSIINTASVGGSIGGCAPHAYTSSKHAVVGLMRNTAIELGPF

GIRVNCVSPYFLATPMVTNFFKLDDGGALDIFSNLKGTNLVPKDVAEAALYLGSDESKYV

SGLNLVIDGGVSVANNGFCVFEQSV*

>MtSDR110C5

MLNSFTRLEGKVALITGGASGIGEATARLFSEHGAQVVIADIQDDKGHSICKELQKSSSS

YVRCDVTKEEDIENAVNTTVFKYGKLDIMFNNAGISGVNKTKILENKLSEFEDVIKVNLT

GVFLGTKHASRVMIPARRGSIINTASVCGSIGGVAPHAYTSAKHAVVGLMRNTAIELGRF

GIRVNCVSPYIVATPLVKKYFKLDDDDDVLDVYSNLKGANLVPKDVAEAALYLGSDESKY

VSGHNLVIDGGFTVVNNGFCVFGESV*

>MtSDR110C6

MATVPSVAAVLRRLEGKVALITGGASGIGEATARLFTEHGAQVIIADIQDDKGYSVCKDL

HKSSASYVHCDVTKEKDIENAVNTTISMYGKLDIMFNNAGIVGANKTNILEYKLSEFEEV

INVNLVGVFLGTKHASRVMIPARRGSIINTASVCGSIGGMASHAYTSSKHAVLGLMRNTA

IELGPFSIRVNCVSPYIVATPMAKNFLKLDDEGLLGLYSNLKGTNLVPKDVAEASLYLGS

DESKYVSGHNLVVDGAVSVSNNGFSVFGQSV*

>MtSDR110C7

MTSVPLVSAALRRLEGKVALITGGASGIGEATARLFSNHGAQVVIADIQDDIGHSICQEL

HKSSATYVHCDVTKEKDIENAVNTTVSKHGKLDIMFNNAGITGINKTNILENKLSEFQEV

IDINLTGVFLGTKHAARVMTPVRRGSIINTASVCGCIGGVASHAYTSSKHAVVGLMKNTA

IELGPYGIRVNCVSPYVVGTPLAKNFFKLDDDGVLDVYSNLKGANLLPKDVAEAALYLGS

DESKYVSGHNLVVDGGLTVGNNGFCIFKQSV*

>MtSDR110C8

MEPRMIQNVESQNLAPKRLLGKVAVITGGARGIGAATAKLFAENGAHVIIADVLDEEGTK

VAESIDGLYIHCDVSKESDIESAINLSISWKGQLDIMFNNAGIAGYEGRSITTLDMEKLT

HLLSINLFGTIHGIKHAAKAMIKGKKGGSIICTSSAAATIGGFASHGYTMSKSAMDGLMR

SAACELGVHLIRVNCVSPHGVPSEMLLNAFRCYGEVDMTSEQLSEFIGMNASLLKGRGAT

TDDVAQAALFLASDESGFVTAHNLSVDGGITSANSVMSFIYQDPK*

>MtSDR110C9

MAEAPSSNTNLRLSGKIAIVTGGASGIGEATARVFANEGVRVVVIADIQDELGNQVAASI

GSQRCTYIHCDVTDEDQVKNLVQSTVDTYGQVDIMFSNAGIASPTDQTIMELDMSQFDRL

FAVNVQGMALCVKHAARAMVEGRIRGSIVCTGSVSCRQGGPRSTDYTMSKHAVLGLMRAA

SVQLAAHGIRVNCVSPSGLATPLTCKLLGMSEEKTQETYQKYARLEGVVLTPKHVADAVL

FLVSDQAEFITGLDLRVDGGFAYGK*

>MtSDR110C10

MAEAPSSNTNLRLSGKVAIVTGGASGIGEATARVFANEGVRVVVIADIQDELGNQVAASI

GIQRCTYIHCDVADEDQVKNLVRSTVDTYGQVDIMFSNAGIVSPTDQTVMELDMSQLDRL

FGVNVRGMALCVKHAARAMVEGSVRGSIVCTGSVSGSVGSSRSTDYTMSKHAVLGLMRAA

SVQLATHGIRVNCVSPNGLATPLTCKLSGMSEEKAQATYQKYARLEGVVLTPKHVADAVL

FLVSDQAEFITDLDLRVDGGFAYGK*

>MtSDR110C11

MAEASSTNSGLRLAGKVAIVTGGASGIGKETAHLFAEQGARMVVIADIQDELGNQVAASI

GSRKCTYIHCDIANEDQVKNLVQSTVNAYGQIDIMFSNAGIASPSDQTILELDISQADHV

FAVNIRGTTLCVKYAARAMVEGRVRGSIVCTASVLGSQGVLRLTDYTISKHAIIGLMRSA

SVQLAKYGIRVNCVSPNGLATPLTMKLLGASAKTVELIYEQNKRLEGVVLNTKHVADAVL

FLVSNESDFVTGLDLRVDGSYVYGKYELL*

>MtSDR110C12

MAESSHTKSSLRLASKIAIVTGGASGIGKETAHVFAEQGARMVVIADIQDELGNEVAASI

GSHRCTYVHCDVTNEDQVKNLVQSTVNTYGQVDIMFSNAGIASPSDQTVLEFDISQADHL

FSVNVRGMALCVKHAARAMVDGCVRGSIVCTASVAGSNGSMKLTDYVMSKHAIIGLMRSA

SKQLAKHGIRVNCVSPNGLATPLTMKLLDAGEETVDLIFGEYKRLEGVVLNTKHVADAVL

FLVSNESDFVTGLDLRVDGSYLDGKSELVF*

>MtSDR110C13

MAEAPSTNTTLRLSGKVAIVTGGASGIGEATARVFANEGTRVVVIADIQDELGNQVAASI

GNQRCTYIHCDVTDEDQVKNLVQSTVNTYGQLDIMFSNAGIISSTAQTIMELDMSQLDRL

FAVNVRGMSLCVKHAARAMVEGHVRGSIVCTGSVGGSRGGSRSTDYTMSKHAVLGLMRAA

SVQLAAHGIRVNSVSPNGLATPLTCKLLGMSNEEAQENYKNYARLEGVVLTPKHVADAVL

FLVSNQAEFVTGLDLIVDGGFAKV*

>MtSDR110C14

MAEAMSRNSSNLRLSGKIAIITGGASGIGEATAHVFANEGASHVVIADIQDELGNQVATS

IGNQRCTYIHCDVADEDQVKNLIQSTVNTYGQVDIMFTNAGIFSPTDQTVLKLDMSQLDR

LFTINVRGMALCVKHAAHAMVEGRIRGSIVCTGSVHSSHGFLRSTDYTMSKHAVLGLMRA

ASVQLAAHGIRVNCVSPNGLATPLTCKLLGVSKEKAQETYKGYARLEGVVLTPKHVADVV

LFLASNDAEFVTGLDLSVDGGFAYGK*

>MtSDR110C15

MKQRLEGKIAIITGAASGIGKDAVKLFVENGAFVVAVDIQDELGHQVVSSIGSNKVEETV

NFTLEKHGQLDVMFSNAGIQGSLLGVLEFDLNEFKKTIDINVIGTAAIIKHAARAMVAKN

VRGSIICTGSVAASTGGTGPSGYTASKHALLGLVRAACSELGGYGIRVNSVSPFGAATPF

ACAPIKIEPEVVEASICSKGNLKGVVLKAKHIAEAALFLASDEAVYISGHNLVVDGGFSV

VHNIEQTV*

>MtSDR110C16

MGVMSEKPLQELSLHSTDTTFSPPSPKRLEGKIAIVTGGARGIGEATVRIFVKHGAKVVI

GDVEDELGIMLANSLSPSAIYVHCDVSVEKDVENLVTSTISHYGKLDIMFNNAGFLGNQS

KNKSIVNFDTEEFDRVMNVNVKGVALGMKHAAKVMIPRGNGCIVSTSSVAGVLGGLGPHA

YTASKHAIVGLTKNASCELGKYGIRVNCISPFGVATSMLVNAWRNGEDEVDEGINFGLPL

IEEVEKMEEFVRGIGNLRGTTLKTQDIAEAVLYLASDESKYVSGHNLVVDGGITSSRNCI

GL*

>MtSDR110C17

MLRTLARELKLTNFSNGLVKKRSSRFYATVGGRRLEGKIAIITGSASGLGKATAHEFVQH

GAQVIIADNDTQLGPKVAKELGHSAQYVECDVTVEAQVEEAVNFAITNYGKLDIMYNNAG

ITGPVIPPSITELDLDEFEKVMRINVTGVIAGIKHAARVMIPKGYGSIICTSSISGLFGG

LGPHPYTISKSTIPGVVKSVASELCGAGIRVNCISPTAIPTPMSLYQIGKFIPGVTYEQI

GEIVSGLSALKGAKCEDIDVARAALYLASDDAKFISGQNLIVDGGFTSIKNFAFPSPDQI

G*

>MtSDR110C18

MASSSPAAVNGRLEGKVALITGGASRIGKRTAEIFVQKGAKVVIADIQDELGHSVAQTIG

SSTCTYVHCDVTDESQIKNVVDTTVQTYGKLDIMFNNAGIGGPNNSRIIDNDKADFERVL

SINVTGVFLGIKHAAQAMIPARTGSIISTTSISSYVGGAASHAYCSAKHAVVGLTKNAAV

ELGKFGIRVNCVSPYALATPLATQFVGCNDGELETTMNTLANLKGVILKTDDVVNAALYF

ASDDSRYVSGHNLLIDGGFSIVNPSFHMFQYSDS*

>MtSDR110C19

MASIGKRLEGKVAIITGGASGIGAATAKLFVQHGAKVIIADIQDEVGQSLCNELGPKNIL

YVHCNVTTESDIKNVVDTAVSNYGKLDIMFNNAGISDDKNREILNYDSEAFKRVFDVNVY

GAFLGAKHAARVMIPNKKGVILFTASVATETAGESTHAYSSSKHALVGLMKNLCVELGQY

GIKVNCISPGAISRD*

>MtSDR110C20

MLRICLRKNLPCSRPLFAESFNRLLSTQTGRKLQDKVALITGGASGIGKAAATKFINNGA

IVIIADIQQQLGQETAKELGPNATFITCDVTKESDISDAVDFSVSEYKQLDIMYNNAGIP

CKTPPSIVNLDLELFDKVMEINVRGVMAGIKHATRVMIPRGTGSILCTASVTGVIGGMAQ

HTYSVSKFAVIGIVKSLASELSGHGIRVNCISPFAIPTPFVMNEMDQIYPHLDSQRLVEI

VRNVGVLKGANCEPNDIANAALYLASDDARYISGHNLVVDGGFTSFKNLEFPAPDQAQ*

>MtSDR110C21

MSRKRLEGKVAIVTGGASGIGAETAKTFVENGAFVVIADINDELGHQVATSIGLDKVSYH

HCDVRDEKQVEETVAFALEKYGTLDIMFSNAGIEGGMSSSILEFDLNEFDNTMAINVRGS

LAAIKHAARFMVERKIRGSIICTASVAASVAGNRGHDYVTSKHGLLGLVRSTCGELGAYG

IRVNSISPYGVATPLACRALNMEMSKVEANMKDSANLKGITLKATHIAEAALFLASEESA

YISGHNLVVDGGFSVINSCVPTTIKK*

>MtSDR110C22

MTKFRLEGKVAIITGAASGIGEETVKLFAENGAFVIAVDIQDELGHKVADSIGSDKVTYH

HCDVRDEKQVEETIHFTLEKHGCIDILFSNAGIIGSLSGILDLDLNEFEKTMATNVVGAA

ATIKHAARAMIAKKIRGSIICTTSVAASIGGTGPNGYTTSKHALLGLVKSACGELGGYGI

RVNSISPFGVATPLSCIAYNLEPHEVESSSSSHANLKGIVLKAKHVAEAALFLASDEAVY

ISGHNLVVDGGFSVVRNTPSAMPDV*

>MtSDR114C1

MGKAEKKEKAKERREKRLEEINLLRSIPYSDHQRWWSKETIAVVTGGNRGIGFEICRQLA

AHGLTVILTSRDASAGAESIKILQEGGLDVVYHRLDIVHESSINHFVEWLQQNYGGLDIL

VNNAGVNFNLGSDNSVENARKVIETNYYGIKKLTEALIPMMKPSVVGARIVNVSSRLGRL

NGRRNRIMNVALREQLSDVEFLSEELIDRTLSTFLQQVEDGSWTAGGWPQIYTDYSVSKL

AVNAYTRLMARKLSERPEGQKIFVNCYCPGWVKTALTGFAGNNTVEEGADTGVWLALLHD

QTVMGSFFAERREINF*

>MtSDR114C2

MESNGRYVAQRYAVVTGANKGIGLEIVKQLAFLGVTVVLTARNDTRGRDAITKLHQTGLS

NVMFHQLDVLDALSIESLAKFIQHKFGRLDILINNAGASCVEVDKEGLKALNVDPATWLA

GKVSNTLLQGVLTQTYKKAEECLNTNYYGVKRVTMALLPLLQLSPAKARIVNLSSLRGEL

KRIPNERLRNELGDVDELSEGKIDAMVKKFLHDFKANDHEANGWGMMLPAYSISKASLNA

YTRVLAKKNPHMLINCVHPGFVSTDFNWHKGTMTVDEGARGPVMLSLLPADGPTGCYFDC

TEIAEF*

>MtSDR114C3

MGHKEKSRKDKRLQEISLLRTIPYSDHQRWWSKETIAVVTGGNRGIGFEISRQLADHGVT

VVLTSRDASVGVESIKVLQEGGLDVHCHQLDILDSSSVNEFAEWLKEEYGGLDILVNNAG

VNSNMGSDNSVENARKCIETNYYGTKRMIEAMIPLMKPSAAGGRIVNVSSRLGRLNGKRN

RIENEELREKLSDVESLSEELIDETINNFLQQIEDGSWKTGGWPQTFTDYSVSKLAVNTY

TRYMAKKLMtSDRPEGEKIYINCYCPGWVKTALTGYAGSVTVEQGADTGVWIALVPDQEITG

KFFAERREINF*

>MtSDR114C4

MEPEPYFPSPALCSTRWWSKETVAVVTGGNKGIGFALVKRFAELGLSVVLTARDKKKGED

AVERIRAQLGLVAPHHHVHFLVLDVSDADSIKTFASSFKDKFGATLDILVNNAAVSFNEL

DENSVDHAENVMKTNFYGPKLLIEALLPLFRCSSSSNSSITRILNVSSRLGSLDKVTNVE

MKRILESDELKEDEINEMVKKFLRDVRNGTWKSQGWPSYWTDYAVSKLALNAYSKVLAKR

YNINTTKLMSVNCFCPGFTQTSMTKGKGTHTADQAASLATILVLLPPHHLPTGKFFLLRN

NTTFQQVANSKL*

>MtSDR114C5

MAEASERVALVTGANRGIGFAICKQLVSNGIKVVLAARDEKRGLEAVDKLKDLALPGHVV

FHQLDVIDPTSIGSFADFLKNQFGKLDILVNNAGIVGAQVDGEALAALGVVVDPSKVDWT

KIYFENYELVEKGLRTNYFGTKELTRILIPLLQCSSSPKIVNVSSSIGRLEILANGRPKE

ILSDVENLTEEKIDEIMNEFLKDYKEGSHETKGWPQSNSAYIVSKVALNAYTRVLAKKYP

SFSINAISPGFVKTDMTHGNGALTSDEGAEPIVKLALQDGSPSGLFFSRGEEKSF*

>MtSDR114C6

MNNAGVPGGIGYETYELAEKCLKTNFYGVERVTEALVPLLQLSTSPTIVNVSSRAGLLKN

ISNDWARKVFNDIDNLTKEKIDEVLKEFEKNYKEGSLEIKDWPTFASAYTMSKAALNAYT

RIMAKKYPHFHINSVCPGFVKTDMNNNIGNLSIDEGVETPLMLALLSNNGPSGCFFTKGE

VIPF*

>MtSDR114C7

MAEAKLRNAVVTGSNKGIGFETVKMLASNGVKVMLTARDEKKGNEAIQKLKQFGLSDQVM

FHQLDVTDPASITSLVEFVKTQFGRLDILVNNAGVSGVNPYETVGSTVDWEKLTQTSDMA

ENCLRTNYYGVKETTEAFLSLLKLSNSPKIINVSSQAALLQNIPNQWAKRVFDDIENLTE

ERIDKVLIEFIKDFKEGSLENKGWPTFLSTYIVSKAATNSYTRILAKKYPNMCINCVCPG

YVKTDLTKNIGMLSVDQGAASVVRLALLPDGSPSGLFFIREEMSNF*

>MtSDR114C8

MAEAKLRYAVVTGSNKGIGFETVKMLASNGVKVVLTARDEKKGNEAIQKLKQFGLSDQVM

FHQLDVTDSASITSLVQFFKTQFGRLDILVNNAGVSGVNPYETVGSTVDWEKLTQTSDMA

ENCLRTNYYGVKETTDAFLPLLKLSNSSKIVNVSSQAALLKNIPNQWAKRVFDDIENLTE

EKIDEVLKEFIKDFKEGSLENKGWPTIMSAYIISKAAMNSYTRILAKKYPNMCINCVCPG

FVKTDINKNTGMLPVDQGAASVVRLALLPDDSPSGLFFIREEISNF*

>MtSDR114C9

MGTEAIRYAVVTGANKGIGYGICKKLASSGVVVVLTARNEERGLEAVERLKNEFDFSDFV

VFHQLDVDDPASVASLASFIKTMFGKLDILVNNAGVPGGKLIDGDALLRKVNGAEIDTKE

IGYETYELAEKCLKTNFYGVERVTEALVPLLQLSTSPTIVNVSSRAGLLKNISNEWARIV

FNDIENLTKEKIDEVLKEFEKDYKEGSLEIKDWPTFASAYTMSKAALNAYTRIMAKKYPH

FHINSVCPGFVKTDMNNNIGNLSIDEGVETPLMLALWSNNGPSGCFFNKGEVISF*

>MtSDR114C10

MNTHTYIYRYAVVTGANKGIGFEICNQLASIGITVIVTARDECRGLQAFDKLKQEFDLAM

SHSDKVIFHHLDVVDPKSISSLANFIKIHFGKLDILVNNAGLLTENEELTEAILKTNYYG

PKGLIKTLFPLLQFSSSPRIVNVSSTVGRLKYFPNGWPKEVVSDVENLTEEKIDKVVNGF

FNDFKEGSLETKGWPLIVSAYSVSKAAININSNTGTLTSYEGAEPIVRLALLPDGSPSGL

FFSRYEELSYNYFNCKIYQSAFAHGIKCIC*

>MtSDR114C11

MAEATTTYAVVTGANKGIGFAVCNQLASKGITVVLTARDEKRGLEAVEKLKHLSLPGLVV

FHQLDVIDAASIRSFVDFIKNQFGKLDILVNNAGITGAEVDGEALVAANIEENGGQIDWS

KIITQTYEQTELGIKTNYYGAKDLTEAFIPLLQLSSSPKVVNVSSSMGKLENLPNGWPKE

VFSDVENLIEEKIDEVLNKFLNDFNEGSLENNGWPINNDMSTYIISKASLIAYTSVVAIK

YPSICINVVCPGFVKTDINYNTGYLTPDEGAESIVNLALLHDGSPSGHFFVRSEEKPF*

>MtSDR114C12

MIADLYAVVTGANKGIGFAVCNQLASKGITVVLTARDEKRGLEAVEKLKQLSLPGLVVFQ

QLDVIDHASIRSFVDFIKNQFGKLDILVNNAGIPGAQVDGEALAAAKIVENGGQIDWSKI

ITQTYEQTELGIKTNYYGAKDLTEALIPFLQLSSSPKVVNVSASMGKLEKLPNGWPKEVL

SDVEELTEEKIDEVLNQFLKDFKEGSLENKGWPDNNLSTYIISKVALNAYTRVVARKYPS

ICINVVCPGFVKTDIIYNTGYLTPDEGAESILRLALLSDGSSGHFFVRNEEKPF*

>MtSDR114C13

MAEATKRYAVVTGANKGIGFAVCKQLASKGITVVLTARDEKRGLEAVEKLKQLSLPGLVV

FHQLDVIDHASIRSFVDFIKNQFGNLDLLVNNAGIPGAQVDGEALAAANTAENGGQIDWS

KIITQTYEETELGIKTNYYGAKDLTEALIPLLQLSSSPKVVNVSSSMGKLEKLPNGWPKE

VLSDIENLTEEKIDEVLNQFLKDFKEGSLVENKGWPINSMSTYIISKASLSAYTRVAAKK

YPSICINAVCPGFVKTDINYNTGYLTPDEGAESILRLALLSDGSSGHFFVRNEEKPF*

>MtSDR114C14

MAEATKRYAVVTGANKGIGFAVCKQLASKGITVVLTARDEKRGLEAVEKLKQLSLPGLVV

FHQLDVIDHASIRSFVDFIKNQFGNLDLLVNNAGIPGAQVDGEALAAANTAENGGQIDWS

KIITQTYEETELGIKTNYYGAKDLTEALIPLLQLSSSPKVVNVSSSMGKLEKLPNGWPKE

VLSDIENLTEEKIDEVLNQFLKDFKEGSLVENKGWPINSMSTYIISKASLSAYTRVAAKK

YPSICINAVCPGFVKTDINYNTGYLTPDEVVTGANKGIGFAVCKQLASKGITVVLTARDE

KRGLEAVEKLKQLSLPGLVVFHQLDVIDHASIRSFVDFIKNQFGNLDLLVNNAGIPGAQV

DGEALAAANTAENGGQIDWSKIITQTYEETELGIKTNYYGAKDLTEALIPLLQLSSSPKV

VNVSSSMGKLEKLPNGWPKEVLSDIENLTEEKIDEVLNQFLKDFKEGSLVENKGWPINSM

STYIISKASLSAYTRVAAKKYPSICINAVCPGFVKTDINYNTGYLTPDEGAESILRLALL

SDGSSGHFFVRNEEKPF*

>MtSDR114C15

MTEASRRYALVTGANKGIGYGICKKLASSGVMVVLTARNEKRGLDAVESLKELGLSDFVV

FHQLDVTDPISVSSLVEFIKIQFGKLDILVNNAGVAGGIVNGENVVKMVRGEISDWNLAL

RQTYELGRKNFMPNEWVRGVFDDIKNVTNEKLGEVLREFLKDYKEGALETKNWPTFVSGY

TMAKAALNSYTRLLALKLPRFRINCLCPDFVKTDINEMKGFLSIDEGAECPVNLALLPDD

GPSGQFFLHDEVISY*

>MtSDR114C16

MTEASKRYALVTGANKGIGYGICKKLASSGVMVVLTARNEKRGLDAVESLKELGLSDFVV

FHQLDVTDPTSVSSLVEFIKIQFGKLDILVNNAGVAGGIVNGENVVKQVRGEISDWNLAL

RQTYELAEECVEINFFGAERVTEALIPLLQLSTSPRIVNVSSRRGKFKFMPNEWARGVFD

DINNVTNEKLGEVLREFLKDYKEGALETKNWPTFVSGYTMAKAALNSYTRLLALKLPRFR

INCLCPDFVKTDINEMKGFLSIDEGAECPVNLALLPDDGPSGLFFLHDEVISY*

>MtSDR114C17

MAISKERYAVVTGSNKGIGLETVKRLASNGIKVVLTARNQKRGIQAFEKLKKEFEFCNLV

VFHQLDVTDPFSIASLVEFVKTQFGRLDILVNNAGINGFNADDMVEPIINWRELSQTYEM

AENCIITNYYGGKETTEAFLPLLQLSDSPVIVNVSSAAGLLKYISNEWARSVLDDTENLT

EELIDEVLKEFLKDFKQGSLENKGWPTYLCAYKLSKAAVNSYTRLLAYRHPNLCINCVCP

GFVKTDMNRNTGDLSVENGAASVVRLALLSSNSTSGNFFARQDLSCF*

>MtSDR114C18

MGETAKRYAVVTGANKGIGFEIVKQLASAGIKVVLTARDEKRGLHALQTLKAYGLSDFVA

FHQLDVADDASVASLADFVKSQFGKLDILVNNAGIIGTIIKDKELINLAIYNRGALSNDN

RRKAMTQTYELAEECLQTNYYGAKITTESLLPLLQLSDSPRIVNVSSTLGQLESIPDGWP

KRFFSEADNLTEEKVDEVLKKFLEDFKNGLLDYDNGWPKTLGAYIISKAAMNAYTRILAK

KFPTICINSVCPGYTITDITANNGLLTVEEGAVSVVKLALIPNGGTSGMFFYRTEVSSF*

>MtSDR114C19

MWVVFVARIAVVTGSNKGIGLEIVRQLASAGIKVVLTARDEKRGLHALETLKASGLSDFV

VFHQLDVANAASVATLADFVKSQFGKLDILVNNAGISGALIDDKDLASLLISNPRALSED

EKKKAVTQTYELAEECLQINYYGAKITTESLLPLLKLSDSPRIVNVSSTTGKLKRIKNEW

TREVFGDVDNLTEEKVDEVLKKFLEDFKEGSMESKGWPKTGGAYVLSKAAMNAYTRILAK

NFPTLCINSICPGYVITDITGNTGLLTAEEGAASVVKLALLPNGSPSGQFYHRTEVMSSF

*

>MtSDR114C20

MGSIAERIAVVTGANKGIGFEIVKQLASAGIKVVLTARDEKRGLHALETLKASGLSDFVV

FYQVDVANAASVATLADFVKSQFGKLDILVNNAGISGTVINDKDLATLLISNPGALTEDE

KKKAVTQTYELAEECLQINYYGAKITTESLLPLLKLSDSPRIVNVSSTLGKLEGIQNEWT

KKVFSDADNLTEEKVDEVLKKFLEDFKEGSLESKGWPKTGGAYVLSKAAMNAYTRILAKN

FPTLCINSICPGYVITDITGNTGLLTAEEGAASVVKLALLPNGSPSGRFYNRTEVSAF*

>MtSDR114C21

MGEPRERYAVVTGANKGIGLEIVKQLASAVIKVVLTSRDEKRGLHALETLKASGLSDFVV

FHQLDVADASSVASLADFVKSQFGKLDILVNNAGIGGVEIKDSDLFTSAIITNGALPDEE

LRRAVTQTYESAKECIQINYYGAKRTFEYLLPLLQLSDSPRVVNVSSGAGKIESVSNEWA

KGVFSDVENLTDERIDEVIKEFIKDFEQGSLERKGWPRFIAPYTIAKASMNAYTRITAKK

YPNFCINCVCPGYVKTDITANTGFFTVEEGAAHPVRLALLPSGSPSGHFYVRNEASSF*

>MtSDR114C22

MGEPTKRYAVVTGSNKGIGFEIVRQLASDGIKVVLTARDEKRGLHALETLKASGLSDFVL

FHQLDVADASSVASLADFVKSHFGKLDILVNNAGISGVEVKDRDLFTSAIMTSGALPDEE

LRRAVTQTYESAKECIQINYYGAKRAFEYLLPLLQLSDSPRVVNVSSFLGKIELVSNEWA

KGVFSDVENLTEERIDEVLEEFIKDFEEGSLESKGWPRFAAAYTVAKASMNAYTIILAKK

YPNFCINCVCPGYVKTDMTTNTGILTVEEGATNPVRLALLPKGSPSGLFYSQNGIASF*

>MtSDR114C23

MGEHRERYAVVTGSNKGIGLEIVRQLASAGIKVVLTARDEKRGLHALETLKASGLSDFVV

FHQLDVADAASVASLADFIKSQFGKLDILVNNAGINGIEIKDSDLYSQVLITNGAQSDEE

LRRTMTYTFESAKECIEINYYGAKRTFEYLLPLLQLSDSPKVVNVSSGLGKIEFVSNEWA
KGVFSDVENLTEERIDEVIKEFIKDFEEGSLERKGWPRYLAAYTVAKASMNAYTRITAKK
YPNFCINCVCPGYVKTDITANTGFFTVEEGAAHPVRLALLPNGSPSGVYYIRNEVYPF*
>MtSDR114C24
MEGEHPDRYAVVTGANKGIGLEIVKQLASARIKVVLTSRDEKRGLHALETLKASGLSDFV
VFHQLDVADAASVASLADFVKSRFGKLDILVNNAGISGVEVNDTDLFSSAIITNGQALSD
EELKTAVTQKFESAKECIQINYHGAKRTFEYLLPLLQLSDSPRVVNVSSFLGKIECVSNE
WAKGVFSDVENLTEERIDEVINEFIKDFEEGSLERKCWPRFAAAYVVGKASMNAYTRIIA
KKYPGFCINCVCPGYVKTDITANTGLFTVEEGAADPVRLALLPNGSPSGLFYSQKEVSSF
*
>MtSDR114C25
MEEYTERYAVVTGANKGIGFEIVKQLASAGIKVVLTARDEKRGLHALETLKASGLSDFVV
FHQLDVADAASVASLAEFVKSRFGKLDILVNNAGIGGIEINDGDLYTKLIMTKGAALSDE
ESRRVITQTLESAKECIQINYYGAKRTFEYLLPLLQLSDSPRVVNVSSRAGTMESSTPCM
EENQRDIGEVSTHKFKSLRSFQDVGMLLDLVNAIERSFEIPLLQLELSLSFP*
>MtSDR114C26
MGHKEKSKEKKDKRSQEISLLRTIPYSDHQRWWSKETIAVVTGGNRGIGFEISRQLAEHG
VTVILTTRDAGVGVESIKVLQEGGLDVACHQLDVLDSSSITQFSDWLKENYGGLNILARI
NFNFGSDNSVENAHTVIDTNYFGTKRMIEAMIPLMKASAAGGRIVNVSTRLGRLNGKRNR
LDNDDLREQLSNVDSLSEELIDGVVTNFLHQVEDGTWKSGGWPRIFTDYSLSKLAVNAYT
RFMAKKLMtSDRPDGEKIFINCYCPGWVKTALTGYAGSVSVEAGADTGVWLSLIPEQAITGK
FFAERREINF*
>MtSDR115E1
MMERRCKVCVTGGAGYIGSLLVKKLLEKGYTVHATLRNLKDESKVSFLRGFPHANTRLVL
FEADIYKPDDFWPAIQGCEFVFHVATPLLHQTDSQFKSIEEAAIAGVKSIAETCIKSRTV
RRLIYTGTVFAASPLKDDGCGYKDYIDETCWTPFQNLHLPLTPFHKDYAYSKTLAERELL
TSYGKDENGSGGFEVVSLVLGLVGGETPLSYLPGSVAVITSQLQDNEALYQSLKFLEDIC
GKCPIVHIDDVCEAHIFCAELPSINGRFLVANSYASSAEIANYYSQNYPEFNLKEKYLEG

PNRAIKLASTKLIDNGFVYKYNLKKILDDSIRCARRTGDLSMS*

>MtSDR115E2

MERKCKVCVTGGAGYIGSLLVKKLLEKGYTVHATLRNLKDESKVGFLKGFPHANTRLVLF

EADIYKPDGFWPAIQGCEFVFHVATPFLHQTDSQFKSIEEAAIASVKSIVETCIKSRTVR

KLIYTGTVVASSPLKDDGCGYKDFIDETCWTPLQSLHLPLTPFHKDYAYSKTLAERELLT

SNGKDENGNGGIEVVTLAVGLVGGDALLSYLPASVAVIISQIHDNEVAYQSLKFLEDIDG

KIPLVHIDDVCEAHIFCAEDPSINGRFLVANSYASSAEIANYYLQNYPEFNLKEKYLEEP

NKAIKWASTKLTDKGFVYKYDLKMILDDSVKCGRRTGDFSM*

>MtSDR115E3

MMERRCKVCVTGGAGYIGSLLVKKLLEKGYTVHATLRNLKDESKVSFLRGFPHANTRLVL

FEADIYKPDDFGTAIQGCEFVFHVATPYLHQTDSQFRSIEEAAIAGVKSIAATCIKSRTV

RRLIYTGTVVAASPLKDDGSGYKDFIDETCWTPLQSLHLPLTDFHKGYVASKTLAERELL

RSYGNDENGSGGFEVVSLVVGVVGGETPLSYLPGSVAVITSQLQDNEALYQSLKFLEDIC

GKIPIVHIDDVCEAHIFCAELPSINGRFLVVNSCASLSEIGNYYSQNYPEFKLKEKYLEG

QNRGIKWDSNKLIDNGFVYKYDLKMILDDNIRCLRRVGDHSMCLVSILEDLK*

>MtSDR115E4

MEARCKVCVTGASGYIASLLINKLLAKGYTVHATLRDLKDESKVGLLKSFPQSQDKLVLF

QADIYNSVDFEPAIKGCEFVFHVATPLIHEPASQFKDITEASLAGSESIAMYCKKAGTVK

RLIYTGSVVSASPRRVDGIGFNDVMDETCWTPLNDSLAYLYHDAYLKDYIYSKTVTEKYM

LSCGNNENGGRLEVVTLLCGAVGGDTLQSFTPGSVAICISHITENAMGRKSLQFVQEFLG

KIPLVHVDDVCEAHIFCMESTSSINGRFLCASSYVSLKEIANHYVLHYPEFTVNQEYADG

PKKDMKWGSTKLCDKGFVYKYDAKMILDDCVKCARRMGDL*

>MtSDR117E1

MGILSISHSTNLLSKIIKIPQNNDWYIPTRRKTRTNVVVFSHQGGGGNVIKSSNLSSVLT

ERSSSLVSSDHAATTLMDAGNLVLSQNGKNQTDIVVKDIVPYGGPTTTTTLIGLDDGIGI

VKFLRGKKFFVTGSTGFLAKVLIEKILRTEPDVGKMYLLIKAKNKQAAMERLQNEIINTE

LFRCLRQIHGKSYQAFMLSKLVPIVGDICETNLGLDEELYDIIADEVDVIVNSAANTTFD

ERYDTAININTRGPCRLMAIAKKCKKLKLFLHVSTAYVNGQRQGRIMERPFSIGDCIARE

KLIPGVPPKYLPTLDIENEINMILKNKGNIEDNLLAQKMKEMGLERARRYGWQDTYVFTK

AMGEMMIDKLREDIPVVVIRPSVIESTLNEPFPGWMEGNRMMDPIVLCYGKGQLTGFLVD

PNGVLDVVPADMVVNATLAAMAKHGTTQKRDINVYQIASSVVNPLVFQDLTRLLYEHYTS

SPCIDSKGNPIQVPIMKLFSSSEEFSGHLWRDAIQKTGLTAMASSNGKMSQKIENICRKS

VEQAKYLAKIYEPYTFYGGRFDNSNTQRLIEMMSEEEKREFRFDVKGIDWKDYITNVHIP

GLRRYVMKGRGMSNQ*

>MtSDR117E2

MELGSILHFLEGRTILVTGATGFLAKIFVEKILRVQPNVKKLYLLLRAKDSESAAQRFRN

EIIGKDLFKLLKENQGPKFNSFVSEKLTLVPGDISKEGLNLKESILEEEICNQTDVIVNL

AATTKFDERYDVALGINTLGVKHVLSFAKKCIQLKVLVHVSTAYVCGERGGLILEDPHRY

GVSLNGVPGLDIDMEKKLVEEKLNQFQAQGTTEHDIEVAMKDLGMERATKYGWPNTYVFT

KAMGEMLVETFKENMSVVIVRPTIVTSTFREPFPGWVEGLRTIDSIVVAYGKGKLTSFMA

DLDAVFDVIPADMVVNAIIVAMMAHANQPNDNIIYHVGSSIRNPITYRTFRDYNLRYFTK

KPLINKDGKSIKVGNITVFSNIASFRRYMFICYMLPLKGLEVANSILCQYFQGIYTDLNR

KISTVMRLIDLYLPYLFFNGIFDDMNTQKLLLAVKQEGVEVNLFYFDPKIIDWEDYFMNI

HIPGIFKYALKF*

>MtSDR117E3

MAQEVGSILNFLEGKTILVIGATGFLAKIFLEKVLRVQPNVKKLFLLLRASDDKSAASRL

QNEILAKDLFNLLNEKMGTNFKSFISEKLTVVPGDITFEDLGLKDSILREDISSQTDVII

NLAATTNFDERYDIALDLNTFGVKHIMSFAKQCIRLKVLVHVSTAYVCGEKSGLILESPY

LLGDSLNGVAGLDINAEKKLVTEKLGELQEKGATEHEIKVAMKDLGITRANVYGWPNTYV

FTKALGEMLVEKLKGNLSVVILRPAIVTSTLREPFPGWVEGLRTIDSLAVVYGKGKLTCF

LGDINGVVDAIPADMVVNAMLVAMVAHANQPRDGVYHVGSSVRNPLRYQSFHDYGLKYFK

AKPWINKDGTVVKVGKMTILTNMASFQRYIFIRYLLPLKGLKLVNTALCQYFQGTYLELN

RKIQVVMRLVELYRPYLFFKGIFDDLNTEKLRMSARQSGTEIDLFYFDPKEMDWEDYFMN

THLPGLVKYIFK*

>MtSDR117E4

MELGSVLHFLQDKTILITGATGFLAKILLEKILRVQPNVKKLYLLLRASDAKSASHRFHN

EIIGKDLFKCLKEKLGANFTTFLSEKLTLVPGDISLEDLGLEDSILKEEIHNQIDVIVNL

AANTNFDERYDISLGLNTFGIKYIINFAKKCNKLKVLVHVSTAYVCGEGEGLILEKPYHL

GHSLNGVNGLDVDIEEKVVRDKLCELQQLGATEDEIKMAMKNLGISRAKLYGWPNTYVFT

KAMGEMLVGQLKGNLSVVIIRPAIVTSTFKEPFPGWSEGVRTIDSLAVAYGKGKLTCFLG

DLNAIVDVIPADMVVNSILVAIVAHANHPNNDAIYHVGSSIRRPLMYSDLQEFGFRHFKA

KPYINKDGKPVKVGKVTVFSNMDSFSRFMFIRYLLMLKGLEIANTALCQYFKGTYLDLKR

KIQIVMRLVDLYKPYLFFKGVFDDMNTEKLRMAARQGGVETDLFYFDPKVIKWDDYFLNI

HLPGVVKYILK*

>MtSDR117E5

MDQFGSIAHFLEDKNVLVLGAAGFLAKIFVEKILRVQPNVKKLYLLLRATDAESATRRMH

NEILRKDLFKLLKENHGAKFNSFISEKVTMVPGDISQENFNLKDSNLLQELYNKTDVIVN

LAATTNFDERYDVALGLNTFGAKHVLNFAKNCINLRVLVHVSTAYVCGERGGLIVEDPYQ

LGVSLNGVQGLDIDEEKRVVEEKLNMLQQEGATEKDTRIAMKDLGMERAKLYGWPNTYVF

TKAMGEMLVGTMKEKLSIVIVRPTIITSTYKEPFPGWVEGVRTIDSLIVAYGKGKLTCFL

ADLEAVFDVIPADMVVNAMLVAMVSHANQPCDDSIYHVGSSVGNPVRYESLRDYCFRYFT

AKPCFDKEGKAIKVGKVTVLENMNSFQRYMYIRYLLPLKGLELVNAAFCRYFQSTCFDIS

RKVHTVMRLVELYRPYVFFNGVFDNMNAEKLQIAARQSGVEMDLFYFDPKMINWEDYFMN

IHIPGIVKYSFK*

>MtSDR117E6

MNSGTMQDFLKGKTILVTGTTGFLAKVFVEKILRIQPDIQKLYLLIRASNTELASHRMQN

EVFQTDLFRVLRDKLGGGFNSFISKKVVAVAGDAAVENLGIKDNTILNVMFEEIDLIVHS

AGTTNFNERFDISMGVNTMGALHVLNVAKKCRKINVLVHISTAYVCGETKEGKPIFQEKP

FEMGRQSLERTLKLDIHTEMNLLEKKLDELRAMNVDEKTIKHALKDYGIERANLHGWPNT

YVFTKAMGEMLLVHHKDNVPLIIIRPTMVTSTSKDPFPGWIEGQRTVDSMICAYGKGKLP

YFLGNPRTILDIMPADLVINCVIAAIVINLNKAPKNFIYHVSSSLRNPLKISDVHNISHQ

YFKKTPCLDEDGKPIVISKGIALKSMAAFNIYTETRYVLPLEVLNLVNKLICHSFQDVYD

DNYKKIRIVKRLAKLYKPYVFFKAVFDDTNTENLRRETMSYNMENGMLEFDPISINWTNY

MMNTHIPGLVKYAMK*

>MtSDR117E7

MNSQDFLKGKTILVTGATGFLAKVFVEKILSIQPEIKKLYLLVRASKTDLAEHRLQNEVF

EIDLFRVLRAKWGEKFSSFISKKVVAIAGDVAVENLGIKDQNILNEIFEEIDLLVHFAAS

TKFDERFDISMGVNTKGPLHAMNIAKNCKRIKAFLHISTAYVCGDAKEGHTLLRETPFKM

DQSLKGTSKLDIHTEMNLLERKLIELKVMNADENTTKWAMKDYGMGRANLHGWPNTYTFT

KAMGEMLLVHHKDDVPLIIIRPTMVTSTSKDPFPGWIEGLRTRRGRLEIGKATYKR*

>MtSDR117E8

MNSQDFLKGKTILVTGAAGFLAKVFVEKILRIQPEIQKLYLLLRASNTDLAENRLRNEVF

EIDLFRVLRAKWGENFSSFISKKVVAIAGDVAIENLGIKDEKLKREIFEEIDLLVHFAAS

TKFDERFDILMAVNTQGALHALNVAKNCKRIKAFVHISTAYVCGDAKDGDSIILRENPFE

MGESLKGTSKLDIHEEMNLLERKLAELQAMNVDENTITCAMKDYGMERANLYGWPNTYTF

TKAMGEMLLVHHKDNVPLIIIRPTMITSTNKDPFPGWIEGLRTIDSLIYAYGHGKVKCFL

GNPKTVIDAIPADMVINCVITAIFIHSSNQRPKNFIYHISSSLRNPLKSSDLHNICHRYF

MKTPCVNQNGKPIIISKGIPVNSFAVFNIYVLVRYVLLLMILNLVNKICRHSFQDVYEKN

SRNLRMLQRLAKLYKPYVFFKSIFDDTNTEILRMATKGYLKMENEEFNFDPTSIDWTDYM

MNTHIPGLIKYQTR*

>MtSDR117E9

MNWGTMQNFMKGKTILVTGTTGFLAKVFVEKILRIQPEIQKLYLLVRASNTDMASHRLQN

EVFDTDLFRVLRDDWGEDFNSFISKKVVAVAGDVAVENLGIKDQNILNVMFEEIDLIVNS

AATTNFDERFDISMGVNTMGALQVLNIAKKCHKVKLLVHISTAYVCGEAKEEGSIFQEKP

FEMGQSLKGTSKLDIHTEMNLLEKKFDELRAMNVDEKTLKYALKDYGIESNGRDLVHHKH

KVPLIIIRPTMITSTSKEPFPGWIEGQRTVDSMICAYGKGKLPYFLGNPRTVLDIIPADL

VINCMIAAIVINSNQAPKNFIYHVSSSLRNPLKISDVHNISHRYFMKTPCLNKDGKPIVI

SKGIAFKSMAAFNIYTETRYVLPLEVLNLVNKLICHSFQDVYVDKNKKIRLVKRLAKLYK

PYVYFKAVFDDTNTKNLRRAVEGYNMENGILEFDPISINWTNYMMNTHIPGLVKYAMK*

>MtSDR117E10

MNSGTIHSFLKGKSILVIGTTGFLAKVFVEKILRIQPEIQQLYLLVRASNNDLASQRLQN

EVFQTDLFGLLRDKLGQEFDSFISKKVTAIAGDVSVQNLGLKDENLNLFQEIDLIVNFAA

TTKFDERFDISMGVNTMGPLHVLNFAKKCCNIKVFVHISTAYVCGEANNGEEILQEKPFE

MGQTLKGTSKLNIQTEMDLLEKKIDELRAMNADESTIKYALKDYGIQRANLHGWPNTYVF

TKAMGEMLVVNQKDNVPLIIIRPTMVTSTNKDPFPGWIEGLRTTDTVIRGYGIGKLACFV

GNPNTILDIIPADLVINCVITTIVVHLDQAPKDFIYHISSSLRNPFKVLDFINIIYDYFV

KNPCTNENGKPIVISKRLFPTSLSGFNVYLTIRYVIPLKVSNYVNKTCFRFSQDATYDDN

YKKNRMLKGWAKLYKPYSCFKAIFDDTNTENLRRVTKSLKENEELNFDPTSIDWTNYMMN

THIPSIVKYAMK*

>MtSDR119C1

MDFLNFLLNLFVPPASLITLAFSWPALCFLNACEWLFNYNYGEDMDSKVVIITGASSAIG

EASCQNQIAYEYAIRRANLMLVARREHRLIGIAENARRMGARHVMIMAADVVKEDDCRRF

VNETINVFGRVDHLVNTVSLGHTFYFEEVTDTSVFPVLLDINFWGNVYPTLVALPYLHQS

NGRVIINASVESWLPLPRMSLFGAAKAALVNFYETLRFELKDEVGVTIATHGWIGSEMTR

GKFMLEEGADMQWKEEREMHVSGEAVEEFARLIVSGACRGDAYVKFPSWYDVFLLYRVFA

PNVLNWAFRLLISPQGTRRFSSYLGTGRSLDTVTGGMGRPMLEGTSPRHHTGMVPLTFSG

QLSHQMQQKMD*

>MtSDR119C2

MSAICNILNVVLPPLSLISIPITMLPYLFVKLLVYAKNLVHTESMERKVVLITGAASGIG

EELAYEYAKRGARLSLIDIRKENLVTVADMARSLGSPDVTIIGADVSKVEDSKRFIDETM

KHFGRLDHLVNNAGVSGIPILIEDIHDLTKYNPIMDTNFWGAVHGTLYAIPHLKNSKGRI

IVVASGCGWFPLPRLSIYNASKAATISFFETLRIELGWSIGITIVTPGFIKTNMALKAYE

EEASLQWIPLGSANECAKDIVKSACRGDMYVTNPSWLKAVFPSKLLFPELVDWAERHIFG

LWQKPSCKNGDLRMSKNNQALKTE*

>MtSDR119C3

MDLIHQFLNIVAPPFTFFSLLLFLPPYWTFKFFLSIIFAFFPENVAGKVVHITGASSGIG

EHLAYEYAKRGARLALSARRDTALREVADRARDCGSPDVIIMRADVSKVDDCRRLVDETV

NHFGRLDHLVNNAAISAAMMFEGVTDITNWRPLMDTNFWGSVYTTRFALTHLRNSRGKIV

VLSSIDSWMPAPRRSIYNASKAALVSLYETLRVEVGADVGVTIVTPGYIESELTKGKVLL

PPEGKMGVDQDMRDVEVSATPVGSVSECAKSIVNGTLRGDRYLTAPAWFRMTYVVKVLCP

ELLEWGFRILYLTRSSNTPAREAPSKKILDATGIKNVFYPSSIRSPEVKTE*

>MtSDR132C1

MGKEEVELEPWQKLDDKVVLVTGASSGLGYDFCLDLAKAGCRIVAAARRLDRLHSLCHQI

NNLYGNGNLLNLRAVAVELDVSADGASIDKSVHKAWDAFGHIDTLINNAGVRGSVKSPLD

LSEDEWNHVFKTNLTGSWLVSKYVCKRICDAHRKGSIINISSTSGLNRGNLSGAVAYASS

KAGLNMLTKVMALELGAHKIRVNSISPGIFKSEITEKLVEKAWLNNVITKINPLRSLVAS

DPGLTSLARYLIHDASEYVTGNNFIVDCGVTLPGVPIYSSL*

>MtSDR132C2

MAKQLEPWHDLAGKVVLVTGASAGLGRDFCLDLAGAGCNVIAAARRVDRLQSLCDEINGK

DGRLRAVAVELDVAADGGAIEKYVQKAWEAFGHIDALINNAGVRGNVSSPLELTEEEWNN

VFRTNITGAWLVSKYVCKLMRDSKRKGSIINISSIAGLERGQVPGGTAYACSKAGVNMLT

KVMALELGAHKIRVNSISPGLFKSEITESLMKKDWLNNVAKRTVPLREWGTSNPALTKIV

RYLIHDSSEYVTGNIFIADAGATLPGFPIYSSL*

>MtSDR132C3

MPKQLEPWHHLAGKVVLVTGASAGIGRDFCLDLARAGCFVIAAARRVDRLQSLCDEINGK

DGRLRAVAVELDVAADGGAIEKYVQKAWEAFGHIDALINNAGVRGNVSSPLELTEEEWNN

VFKTNITGTWLVSKYVCKLMRDSKRKGSIINISSIAGLERGQLPGGTAYACSKAGVNMLT

KVMALELGAYKIRVNSISPGLFKSEITESLMKKDWLNNVAIRTVPLREWGTSNPALTSIA

RYLIHDSSEYVTGNIFIADAGATLPGFPIYSSL*

>MtSDR132C4

MVLDHTRSVYYTLYCQLHWLHFVKTICSARMLYCIKGSRHDDFLLTTDDHGNVSSPLELT

EEEWNNVFRTNITGAWLVSKYVCKLMRDSKRKGSIINISSIAGLERGQVPGGTAYACSKA

GVNMLTKVMALELGAHKIRVNSISPGLFKSEITESLMKKDWLNNVAKRTVPLREWGTSNP

ALTKIVRYLIHDSSEYVTGNIFIADAGATLPGFPIYSSL*

>MtSDR132C5

MPKQLEPWHHLAGKVVLVTGASAGIGRDFCLDLARAGCFVIAAARRVDRLQSLCDEINGK

DGRLRAVAVELDVAADGGAIEKYVQKAWEAFGHIDALINNAGVRGNVSSPLELTEEEWNN

VFKTNITGTWLVSKYVCKLMRDSKRKGSIINISSIAGLERGQLPGGTAYACSKAGVNMLT

KVMALELGAYKIRVNSISPGLFKSEITESLMKKDWLNNVAIRTVPLREWGTSNPALTSIA

RYLIHDSSEYVTGNIFIADAGATLPGFPIYSSL*

>MtSDR152C1

MASLTGSNCVALRSATFAATGNRKITQIRHYSPLLNHPRLVSGLHSRSNTSFNSTGLRAQ

VATLAEASTEAVQKVESPVVIVTGASRGIGKAIALALGKAGCKVLVNYARSSKEAEEVSK

EIEALGGQALTYGGDVSNEADVNSMIKTAVDAWGTIDVLINNAGITRDGLLMRMKKSQWQ

EVIDLNLTGVFLSTQAAAKIMMKNKKGRIINISSVVGLIGNAGQANYAAAKAGVIGLTKS

VAKEYSSRGITVNAVAPGFIASDMTAKLGNDLEKKILEAIPLGRYGQPEEVAGLVEFLAL

SQAASYITGQVFTIDGGMVM*

>MtSDR357C1

MAGSDGDRGGGKGIEMGIEIGRSEGNRIVIKTNVSKGLGRVLVIELLAYRSHTIIGCSCD

QDKLDFLQSQLPNNNHHLFLNIDVRCNNRVEEMACIFMEKNGGPSDIIVNGAGVVNKNNK

MWEVPSEEFDLVMDTNLKGAANVLRPFIPLMVKNKKYEEGGIIVNMSSGWGRSVAALVAP

YCVSKWAIEGLTKSVVEELPKVMAVVALNPGVINTNMLAFCYGASSSLYRFPESWVLEAA

TKILNLTPTDNGSSLSI*

>MtSDR357C2

MVGSDGERGGGKGIEIGIGIGRSKGNRTVMITGVSKGLGRALAIELAYHDHTIIGCSDEE

MACIVMEKNGGPPNIIVNDAGVINKNNKMWEVLSEEFDLVMDTNLKGNVNVLRHFISLMV

KKNKNEEGGIRSGAALVAPYFASKWEIEGLTKSVAEEFPKGMAVMALNTGVISTNILASC

YGASSSLYQSPESWVLEEATKILNLTPTNNGASLSI*

>MtSDR357C3

MGGKGIGNKSVLITGVSKGIGRALAIELANRGHTIIGCSRAQDKLDSLQSLLLPNNHNHL

FLNVDVSSNDSVQQMARTVMEMKGGPPDVIVNSAGTINKNNKMWEVPSQEFDLVMDTNLK

GTANVLRHFIPLMINNGGGEGIIVNLSSGWGRSGAALVAPYCASKWAIEGLTKSVAKELP

QGMAVVALNPGVINTDMLASCFGASASLYQSPESWALKAATMILNLTPADNGASLSV*

>MtSDR358U1

MAGSSQTTVLVTGAGGRTGQIVYKKLKEKRDQYIARGLVRSEESKQKIGGADDIFLGDIR

NAESIVPAIQGTDALIILTSAVPQMKPGFDPTKGGRPEFYFDDGAYPEQVDWIGQKNQID

AAKAAGVKHIVLVGSMGGTNPNHPLNSLGNGNILVWKRKAEEYLSNSGVPYTIIRPGGLL

DKEGGVRELIVGKDDELLQTETKTIPRADVAEVCVQVLNYEETKLKAFDLASKPEGAGEP

TKDFKALFSQLTSRF*

>MtSDR358U2

MLIMATTTRVPFVSATTFFPNQCHKYSLVARTINLPVSSTSLRLSSCYSTSLVSLALPRS

FKRGGNRRSVVVVAMAESSKSTVLVTGAGGRTGQIVYKKLKERPNEYIARGLVRSEESKQ

KIGAADDVFIGDIRDTESLAPAIQGIDALIILTSGVPLMKPGFDPTQGKRPEFYFEDGAY

PEQVDWIGQKNQIDAAKAAGVKQIVLVGSMGGTDLNHPLNSLGDGNILVWKRKAEQYLAD

SGIPYTIIRAGGLQDKEGGIRELVIGKDDELLKTDIRTIARPDVAEVCLQALNFEEAQFK

AFDLASKPEGTGSPTKDFKALFSQITTRF*

>MtSDR368C1

MKKLLVTVTNNNIYTMEKINSNDQEDSKPVVLITGCSGGGIGNALARSFAANSCNVVATS

RSRSTMADLDQDPKFFLQELDVQSDESVNRVVNTVLDKFGRIDVLVNNAGVPCTGPLAEV

PLSAIQNTFNTNVFGSMRMVQAVVPHMATRKQGKIVNVGSVTALASGPWGGTYSASKAAL

HALTDTLRLELGHFGIDVVNVVPGAVKSNMGNSGIAIYDRMPEWKLFKPFEAVIRERALL

SRKLRQTPTDEFAKHTIAAILRKKPPAWFSYGNYSTVMAIMYYLPLCVRDFLLKKAMK*

>MtSDR368C2

MEKINSNDHEDSKPVVLITGCTGGGIGNALARSFAANSCRVVATSRSRSTMADLDQDPKF

FLQELDVQSDESVNRVVNNVLDKFGRIDVLVNNAGVPCVGPLAEIPLSAIQNTFETNVFG

SMRMVQAVVPHMATRKRGKIVNIGSVTGLVSGPWNGAYCASKAALHALTDTLRLELGHFG

IDVVNVVPGAVKSNIGNSGIAIYNRMPEWKLFKPFEAAIRERALLSQGLRSTPTDEFARH

TVAAVLRKKPPAWFSYGRYSTAMAVMYHLPLCVRDFLFKKAMKC*

>MtSDR368C3

MSEQKIVLVTGCAKGGIGYEYCKAFAEKNCRVIASDISSRIKDMSDFESDNNIETLELDV

SSDQSATSAVDTIISKYGRIDILVNNAGIGSTGPLAELPLDTIRKTLEINTLGQLRMVQQ

VVPHMALKKSGTIVNVGSVVGNISTPWAGSYCASKSAIHAMSNSLRLELKPFGINVVLVM

PGSIRSNLGKANLEKLSDYDWKLYKDFKEAISERARASQGGKATDGRVFARHVVNKILAN

KPPKQIIFGHLTGLFALLSWSPLWVRDMFFSSRFGLDRKV*

>MtSDR369C1

MATATTTSSSSNEPLLKKKKKQSLGWIEWLRGWFNLFYEFLFQRITASHLHNPMPLPPIN

DLTCIVTGSTSGIGLEIARQLAESGAHVVMAVRNTKRANDLIQKWQIESDGLGLGMALNV

EVMEVDLLSLDSVARFAEAWNARATPLHALINNAGIFSIGEPQKFSKDGYEDHLQVNHLA

PALLSILLLPSLIRGSPSRIVNVNSIMHHVGFVDTEDMNLTSGKRKFSSLVGYSSSKLAQ

VMFSSVLFKRLPAEAGISVLCVSPGIVQTNVARDLPKSVQTGYHLIPYFIFNAQEGSRST

LFAATDPQVSEYCELLKSDEWPVCPYISHDCRPANASEEAHNLQTSHEVWEKTLEMIGLP

SDAVEKFLEGEEVKCRYGQEQQ*

>MtSDR460A1

MEANKSRILVFGGTGYIGKYMVKASISLGYPTLVYTRPINSQTPTSKIQLCKEFSSIGVT

LVEGELEHNQIVAVIKQADIVICTFAYPQVMEQLKIIEAVKVAGNIKRFLPSDFGVEEDR

VKPLPPFQGFLDKKRKIRREIEASGIPYTFVSANCFGAYFVNFLLHPYENKKDIMVYGTG

ETKAVLNYEEDVAMYTIKVANDPRAHNRIVVYRPLKNFITQNELISLWELKNGQILNKVF

APEEDIVKLSQILPPPHNIPVSILHSVFVQGDLVNFELEEDDLEASQLYPNYNYMSIDQL

LDKFLVDPPPPASASFE*

>MtSDR460A2

MEANKNRILVFGGTGYIGKYVVKASISLGYPTLVYTRPINSQTSPSKIQLCKEFNSIGAT

LVEGELEHDQIVRVIKEADIVICTFPYPQVMEQLKIVDAIKVAGNIKRFVPSDFGVEEDR

VHPLPPFQAFLDKKIKIRREIEAAGIPYTYVSANCFGAYFVNILLRPYEKNKDIVVHGSG

QVKAVLNYEEDVAMYTIKVANDPRTHNRIVVYRPSKNIISQNELISLWELKSGQKFHKVF

VPEEDIVKLSQTLPPPEDIPISIIHSIFVRGDMANFELEEDDLEASQLYPGYNYTSIDQL

LDKFLVDPIPPAYGAFE*

>MtSDR460A3

MEAKKNKILVFGGTGYIGKYMVKASISLGHPTFVYTHPINSKTPNSKIQLCKEFNSIGVT

LVEGELEHDQIVKVIKQVDIVICTFPYPQVLEQLKIIDAIKVAGNIKRFLPSDFGVEEDR

VHPLPPFQAFLDKKIKIRREIEAAGIPYTFVSANCFGAYFVNFLLRPYENKKDIVVYGSG

ESKAILNYEEDIAMYTIKVANYPRAHNRIVVYRPLKNIISQNELISLWELKSGQNFNKVF

VPEEDIIKLSQTLPPPEDIPISIVHSIFVKGDMYFELEENDLEASQLYPNYNYTSIDQLL

DKFLVDPPPPVSAAFE*

>MtSDR460A4

MAEKSKILFIGGTGYIGKHIVEASAKAGHPTFALVRESTLADPAKANLLNNFKTLGVNLV

PGDLYNHENLVKAIKQVDVVISTVGHAQIEDQVKIIAAIKEAGNVKRFFPSEFGNDVDRV

HAVDPAKSAFEGKARIRRAIEAEGIPYTYVSSNYFAGYFLPTLAQPGQFAPPPPKDKVVI

YGDGNPKAVFNKEDDIGTFTIRAVDDPRTLNKILYIKPPKNIISFNELVALWEKKIGKTL

EKTFLLEDKLLKDIAEAPFPINVVLSINHSVFVKGDHTNFVIEPSFGVEAYELYPDVKYT

TVEEYLDQFV*

>MtSDR460A5

MAVKSRILIIGGTGYIGKHIVEASVKVGHDTFALVRESTMADPTKAKLLHNFKTIGVNLV

HGDLYDNESLVKAMKQVDVVISALSHTHAADQLKIIHAIKEAGNVKRFFPSEFGNDVDRV

HAVEPAKSVFSVKAQIRRMIEAEGIPYTYVSTNSFAGYFVPTFVQPGATGPPTDKVIILG

DGNKKAVFNKEEDIGTYTIKAVDDPRTLNKILYLRPPKNIYSFNDVIALWEKKIGKTLEK

IYVSEDKLLRDIEEKPFPDNVGLAICHSVFVKGDHTNFEIEPSFGVEASALYPDVNYTTL

EECLDHFI*

>MtSDR460A6

MAGGSNMMSKILLIGGTGYIGKFIVEASAKAGHPTFLLIRESTLSNPTKSSIINKFKDLS

VNFVLGDLYDHQSLVKAIKQVDVVISTVARSHLSDQDKIISAIKEAGNVKRFFPSEFGND

VDRSHAVEPAKSAYAVKARIRRSIESEGIPYTYVSSNYFAGYFLPSLSQHGASAPPRDKV

VILGDGNPKAVFNKEEDIATYTIKSVDDPRTLNKILYIRPQGNALSFNDLVSLWEKKIGK

TLERIYVPKEQLLKQIQESSPPLNMMLSIAHCVYIKGDHTNFEIDPTFGVEATTLYPDVK

YTTVDEFLNQFV*

>MtSDR460A7

MAIGATTKILVIGGTGYVGKFIVEASIKAGYPTFALIRASTLSNPHKSSIIQYFNALGVN

IVLGDIYDHQSLVKVIKQVDIVISSVNHEHISDQYKILAAIKEVGNIKRFFPSEFGNDVD

RNHGVNEGKLVFDTKAKFRRAIEDEGIPHTYVVANFLTRHFLPTKSQLNDTTFPLDTVII

LGDGNTKAIFNTEESVAAFTIRTIDDPRTLNKILYLRPSTNTLSYNDLVSLWEKKTNNNL

KRIYIPEKQVLKMIQESPYPVNMGLAICLAAYVNGDHTNYEIDPSTGVEASELYPDVKYI

TLDQYFEENHDRTPFYLNWLLSLNKEQHFN*

>MtSDR460A8

MAVAVKSTNVLVIGGTGSVGKFIIEASVKAGHPTFALVRESTMFNPAKSPIIQTFKNLGV

NLVLGDIHDHESLVKAIKQVDVVISTVSYLHIPDQYKIISAIKEAGNVKRFFPSEFGNDV

DRSNGVNWAENLFNNKAQIRRTIEVEGIPHTFVVANFFAGHFLPNLSGLRALLTPTNKVI

IFGDGNPKAVFNTHEDVATYTIQAIDDPRTLNKILYVRPHANTISFNELVSIWEKNTSNT

LERVYVPEELILRQIQESSFPNTMSLSICHATFVKEDQTNFEIEPSFGVEASQLYPHVKF

TTIDEFLERNIDRTPFYLNQLIPENIE*

>MtSDR460A9

MAAEKSKILVLGGTGYIGKFIIEASAKAGHPTFALIRESTVSHPEKSKLIESFKTSGVTL

LYGDLGDHESLVKAIKQVDVVISTLGGAQVDDQVKLIAAIKEAGNIKRFLPSEFGIDVDR

HHAVEPVASFFGQKAKIRRAIEAEGIPYTYISSNAFAGYFLPTLGQQNVTSPPRDKVVIL

GDGNVKGVYVTEEDIGTYTIKAVDDPRTLNKVVYFRPPANVLSFNELVSLWENKIKSTLE

KIYVPEDQLLKSIQESPFPANLMLALGHSMSVKGDCTNFEIEPSFGVEASEIYPEVKYTT

VDNYLNAFV*

>MtSDR460A10

MATENKILILGPTGAIGRHIVWASIKAGNPTYALVRKTPGNVNKPKLITAANPETKEELI

DNYQSLGVILLEGDINDHETLVKAIKQVDIVICAAGRLLIEDQVKIIKAIKEAGNVKKFF

PSEFGLDVDRHEAVEPVRQVFEEKASIRRVIEAEGVPYTYLCCHAFTGYFLRNLAQLDVT

DPPRDKVVILGDGNVKGAYVTEADVGTFTIKAANDPNTLNKAVHIRLPKNYLTQNEVISL

WEKKIGKTLEKTYVSEEQVLKDIQESSFPHNYLLALYHSQQIKGDAVYEIDPTKDIEASE

AYPDVTYTTADEYLNQFV*

>MtSDR460A11

MATENKILILGPTGAIGRHIVWASIKAGNPTYALVRKTPGNVNKPKLITAANPETKEELI

DNYQSLGVILLEGDINDHETLVKAIKQVDIVICAAGRLLIEDQVKIIKAIKEAGNVKKFF

PSEFGLDVDRHEAVEPVRQVFEEKASIRRVIEAEGVPYTYLCCHAFTGYFLRNLAQLDVT

DPPRDKVVILGDGNVKGAYVTEADVGTFTIKAANDPNTLNKAVHIRLPKNYLTQNEVIAL

WEKKIGKTLEKTYVSEEQVLKDIQESSFPHNYLLALYHSQQIKGDAVYEIDPTKDIEASE

AYPDVTYTTADEYLNQFV*

>MtSDR460A12

MGKSKVLVVGGTGYIGRRIVKASLEQGHETYVLQRPDIGLETEKVQMLLSFKKLGAHLVE

GSFSNHQSLVDAVKLVDVVICTMSGVHFRSHNLMLQLKLIEAIKDAGNVKRFLPSEFGMD

PALMGHALEPGRVTFDEKMTIRKTIEDANIPFTYISANCFAAYFAGNLSQMGTLFPPRDK

VVLYGDGNVKVVYMDEDDVATYTIKTIDDPRTLNKTIYIRPPENILTQRELIEKWEKIIG

KQLEKSTISEQDFLSSMKGLDLASQVGVGHFYHIFYEGCLANFEIGDGEEASKLYPEVQY

TRMDEFLKLYA*

>MtSDR460A13

MGKSKVLVVGGTGYIGRRIVKASLEQGHETYVLQRLDIGLETEKVQMLLSYKKLGAHLVE

GSFSNHQSLVDAVKLVDVVICTMSGVHFRSHNLMLQLKLIEAIKDAGNVKRFLPSEFGMD

PALMGHALEPGRVTFDDKMAIRKAIEDAKIPFTYISANCFAGYFAGNLSQMGTLFPPRDK

VVLYGDGNVKVVYMDEDDVATYTIKTIDDPRTLNKTIYIRPPENILTQRELIEKWEKLIG

KQLEKSTISEQDFLSSMKGLDLAGQVAVGHFYHIFFEGCLTNFELEDGEEASKLYPEVQY

TRMDEFLKAYA*

>MtSDR460A14

MEKSKVLVVGGTGYIGRRIVKASLEQGHETYVIQRPELGLQIEKLQRLLSFKKQGAHIVE

ASFSDHKSLVDAIKKVDVVISAISGVHIRSHSIGLQLKLVDAIKEAGNIKRFLPSEFGLD

PARMGHALEPGRVTFDDKMAVRKAIEEANIPFTYISANLFAGYFAGSLSQMGSFVPPRDK

VHLFGDGKHKAIFLDEYDVATYTIKTIDDPRTLNKTLYLRPQENILSQGELIGIWEKLIG

KDLEKTYIPPEGFLTTLKGLEYKLQVAIGHFYHIFYEGCLTNFEIGEDGEEASKLYPEVN

YTRMDEYLKIYV*

>Medtr3g101500.1

MKKVVVTGASGYLGGKLCNSLHRQGYSVKVIVRPTSNLSALPPSTEIVYGDITDFSSLLS

AFSDCSVVFHLAALVEPWLPDPSKFITVNVEGLKNVLEAVKQTKTVEKLVYTSSFFALGP

TDGAIADENQVHHERFFCTEYEKSKVATDKIALQAASEGVPIVLLYPGVIYGPGKVTAGN

VVAKMLVERFSGRLPGYIGKGNDKFSFSHVDDVVEGHIAAMKKGQIGERYLLTGENASFN

QVFDMAAVITNTSKPMVSIPLCVIEAYGWLLVLISRITGKLPFISPPTVHVLRHRWEYSC

EKAKMELDYKPRSLREGLAEVLIWLKNLGLVKY*

>Medtr4g086400.1

MEIKAGLSALVTGGASGIGKGLVLALAEKGVFITIVDFSEEKGRETATLVEKINTKFHPN

LHHPSVLFVKCDVTNSRDLAAAFEKHVSTYGGLDICIASAGINNPIPFDKDPTDGTRSWR

HTLNVNFIAVFDTTRLAIKAMEALKRPGTIINLGSASGLYPMYGDPVYTGSKGGVVMFTR

ALRLYKRQGIRINVLCPEFIETEMGLKVDPRLISMMGGFVPMDMVVKGAFELITDESKAG

HCLWITNRRGLEYWPTPSEEAKYLTRPTRFRRKSEYQAPSIKLPDSFEKTVVHTLTHNFR

NATSIVRAPLRLPIKPNHVLVKIIYAGVNASDVNFSSGRYFGGNNKETAARLPFDAGFEA

VGIIAAVGDSVTDLKVGMPCAFMTFGGYAEFTMIPSKYALPVPRPDPEAVAMLTSGLTAS

IALEKAGQMESGKVVLVTAAAGGTGQFAVQLAKLAGNTVVATCGGGAKAKLLKELGVDRV

IDYHSEDIKTVLKKEFPKGIDIIYESVGGDMLKLCLDALAVHGRLIVIGMISQYQGEKGW

TPSKYPGLCEKLLSKSQAVAGFFLVQYSHMWQEHLDRLFDLYSQGKLKVAVDPKKFIGLH

SVADAVEYLHSGKSAGKVVVCVDPTFSNHAAKL*

>Medtr4g086410.1

MEIKAGLSALVTGGASGIGKGLVLALAEKGVFITIVDFSEEKGRETATLVEKINTKFHPN

LHHPSVLFVKCDVTNSRDLAAAFEKHVSTFGGLDICIISAGIENPIPFDKDQTDGTRSWR

HTLNVNFIAVFDTTRLAIKTMEALKRPGAIINMGSASGLYPMYLDPIYSGSKGGVVMFTR

SLRLYKRKGIRVNVLCPEFVETELGLKVDPKFLSMMGGFIPMEMVVKGAFELITDESKAG

HCLWISNRRGLEYWPTPSEEAKYLVRPRRLRRRAEYKAPSIKLPESFEKIVVQTLTHNFR

NATSIVRAPLRLPVKPNLVLVKIIYAGVNASDVNFSSGRYFGGNNKETTARLPFDAGFEA

VGIIAAVGDSVTDLKVGMPCAFMTFGGYAEFTMIPSKYALPVPRPDPEGVAMLTSGLTAS

IALEKAGQMESGKVVLVTAAAGGTGQFAVQLAKLAGNTVVATCGGGTKAKLLKELGVDRV

IDYNSEDIKTVLRKEFPKGIDIIYESVGGDMLKLCLDALAVHGRLIVIGMISQYQGEHGW

TPSKYPGLLEKLLAKSQTVAGFFLVQYSHFYQEHLDRLFDLYSKGKLKVAVDPKKFIGLH

SVADAVEYLHSGKSVGKVVVCVDPTFVNQVAKL*

>Medtr6g088500.1

MATTIVKLQVFPECNLNKHKLRNGFGSSNSSSGVFGFGQNFGGLCLKKCRAFKSEDGGDV

KEKKLRNLKKNEVKVQRENGFWSNFRNVLLGNFMMGSKLDDEYRQAVVRVDEVLSKIAVQ

IGRYIVTMMSTGVILAIGFQMSGGDSQMDALIWYSWLGGVIIGTMIGANWVLEDYCREGP

RNVVITGSTRGLGKALAREFLLSGDRVIVTSRSPESVQATVKELEENLKEGIANAVGSSL

TKLSQAKVVGIACDVCEANDVQRLANFAVSELGYIDIWINNAGTNKGFRPLLQFSDEDIK

QIVSTNLVGSILCTREAMRIMRNQTKPGHIFNMDGAGSGGSSTPLTAVYGSTKCGLRQFH

GSLLTECKRSKVGVHTASPGMVLTELLLSGSTIQNKQMFNIICELPETVARTLVPRMRVV

KGTGKAINYLTPPRILLALVTAWLRRGRWFDDEGRALYSAEADRLRNWAENRARFSFTDA

MEMYTENTWLSVFSLSVVCAFIILSSTSSNLPGT*

>Medtr8g044240.1

MASSMASFQLLSSHQLCISPFSRLPKFNTTFTSCTPNSNNNNRFSLSLTKPTSSSFLIIT

ASSNDNTMLPPYNVLITGSTKGIGYALAKEFLKAGDNVLVCSRSDERVETAVKSLREEFG

EQHVWGTTCDVKNGEDVKKLVSFAKEKLKYIDIWINNAGSNAYSYKPLAEASDEDLIEVV

TTNTLGLMICCREAIKMMVGQPRGGHIFNIDGAGSDGRPTPRFAAYGATKRSVVHLTKSL

QAELQMQDVKNVMVHNLSPGMVTTDLLMSGANTKQAKFFINVLAEPAEVVAEYLVPNIRS

IPANRSMKPTYIRFLTGLKAYSQIFSRFAFGARRNRYIIED*
