# Supplementary figures and images for: Genome-Wide Identification and Characterization of Short-Chain Dehydrogenase/Reductase (SDR) Gene Family in Medicago truncatula
Source: Int J Mol Sci. 2021 Aug 31;22(17):9498. doi: 10.3390/ijms22179498 (PMC8430790; doi:10.3390/ijms22179498)

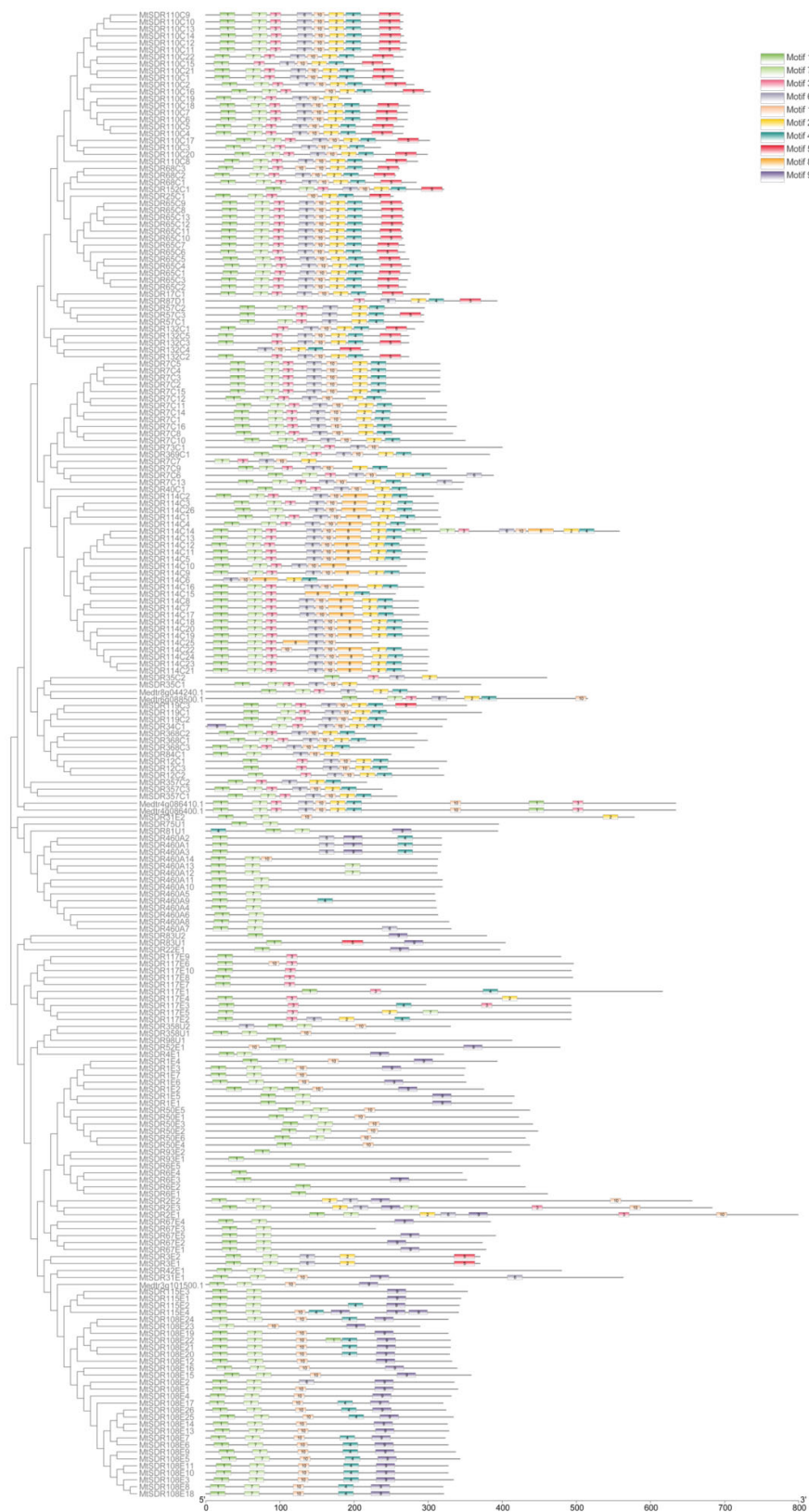

Supplement: Supplementary file 1 [file ijms-22-09498-s001.zip › Figure S1.pdf]
